# New Sesquiterpenoids from the Mangrove-Derived Fungus *Talaromyces* sp. as Modulators of Nuclear Receptors

**DOI:** 10.3390/md22090403

**Published:** 2024-09-03

**Authors:** Tanwei Gu, Jian Cai, Danni Xie, Jianglian She, Yonghong Liu, Xuefeng Zhou, Lan Tang

**Affiliations:** 1 NMPA Key Laboratory for Research and Evaluation of Drug Metabolism, Guangdong Provincial Key Laboratory of New Drug Screening, School of Pharmaceutical Sciences, Southern Medical University, Guangzhou 510515, China; nanyigtw@163.com (T.G.); hc2000822@163.com (D.X.); 2 CAS Key Laboratory of Tropical Marine Bio-Resources and Ecology/Guangdong Key Laboratory of Marine Materia Medica, South China Sea Institute of Oceanology, Chinese Academy of Sciences, Guangzhou 510301, China; caijian19@mails.ucas.ac.cn (J.C.); shejianglian20@mails.ucas.ac.cn (J.S.); yonghongliu@scsio.ac.cn (Y.L.); 3 University of Chinese Academy of Sciences, Beijing 100049, China

**Keywords:** mangrove-derived fungus, sesquiterpenoids, talaroterpenes, nuclear receptors

## Abstract

Four new sesquiterpenoids, talaroterpenes A–D (**1**–**4**), were isolated from the mangrove-derived fungus *Talaromyces* sp. SCSIO 41412. The structures of compounds **1**–**4** were elucidated through comprehensive NMR and MS spectroscopic analyses. The absolute configurations of **1**–**4** were assigned based on single-crystal X-ray diffraction and calculated electronic circular dichroism analysis. Talaroterpenes A–D (**1**–**4**) were evaluated with their regulatory activities on nuclear receptors in HepG2 cells. Under the concentrations of 200 μM, **1**, **3** and **4** exhibited varying degrees of activation on *ABCA1* and *PPARα*, while **4** showed the strongest activities. Furthermore, **4** induced significant alterations in the expression of downstream target genes *CLOCK* and *BMAL1* of RORα, and the in silico molecular docking analysis supported the direct binding interactions of **4** with RORα protein. This study revealed that talaroterpene D (**4**) was a new potential non-toxic modulator of nuclear receptors.

## 1. Introduction

Nuclear receptors (NRs) are an important class of drug targets because they are key regulators of many cardiovascular, metabolic and inflammatory diseases [1]. PPARs (peroxisome proliferator activated receptors), FXRs (farnesoid X receptors, FXRs), LXRs (liver X receptors) and RORs (retinal acid receptor-related orphan receptors) are important members of the nuclear receptor family, playing critical roles in regulating fat synthesis, cholesterol metabolism, fatty acid oxidation, inflammatory response and so on. In recent years, considerable research efforts have been devoted to the development of agonists or inhibitors targeting those receptors. Our previous review summarized 261 natural LXR/FXR modulators, originating from terrestrial plants and microorganisms, marine organisms and marine-derived microorganisms, in the recent two decades (2000–2020) [2]. Obeticolic acid is an FXR agonist that inhibits downstream CYP7A1 expression [3], suppresses bile acid synthesis, promotes bile secretion and so reduces intracellular bile acid concentration. It has been approved by the FDA for the treatment of primary biliary cirrhosis. Saroglitazar, a new PPAR agonist [4], which shows an evident effect on fat metabolism and insulin resistance, has been approved in India for two indications, including diabetes dyslipidemia and type 2 diabetes that cannot be controlled by statins.

Marine microorganisms produce metabolites with unique chemical structures that could target specific cellular physiological and biochemical processes, thus exhibiting significant biological activity and medicinal value [5,6,7]. In our previous studies, we discovered several LXRα agonists from marine microorganism-derived natural products [8,9,10,11], and some of them could promote lipid efflux by upregulating *ABCA1* expression and reducing lipid synthesis, which could be considered as lead compounds for hypercholesterolemia [12]. 

Mangrove sediment-derived microbes, as a rich reservoir of natural product diversity, could be utilized in the research and development of new drugs [13], and many lead compounds have been obtained through our extensive research [14,15]. In our ongoing pursuit of pharmaceutically active molecules, especially natural modulators of nuclear receptors, regulatory activities on nuclear receptors were tested after the natural products were obtained from the mangrove sediment-derived microbes.

**Figure 1:**
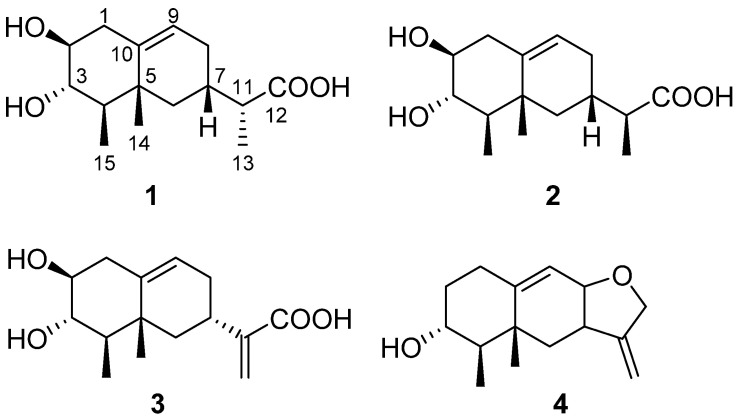
Structures of compounds **1**–**4**.

In this study, four new sesquiterpenoids, talaroterpenes A–D (**1**–**4**), were isolated from a mangrove sediment-derived fungus *Talaromyces* sp. SCSIO 41412 (Figure 1). Herein, the specifics of the isolation, structural elucidation and bioactive assessments of isolated compounds are reported.

## 2. Results and Discussion

### 2.1. Structural Determination

**Figure 2:**
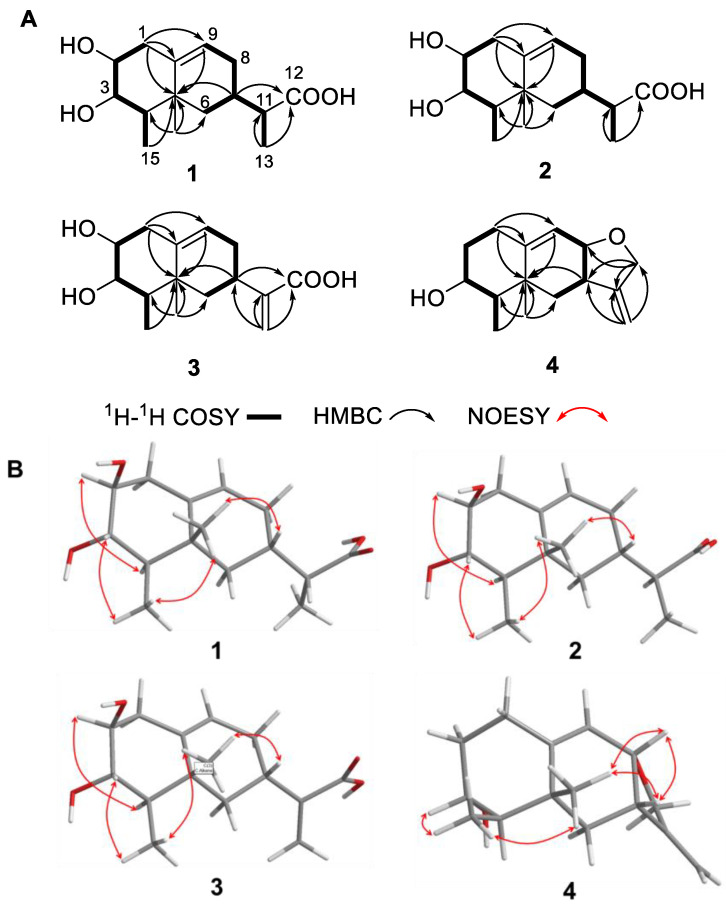
Key ^1^H-^1^H COSY, HMBC (**A**), and NOESY (**B**) correlations of **1**–**4**.

**Figure 3:**
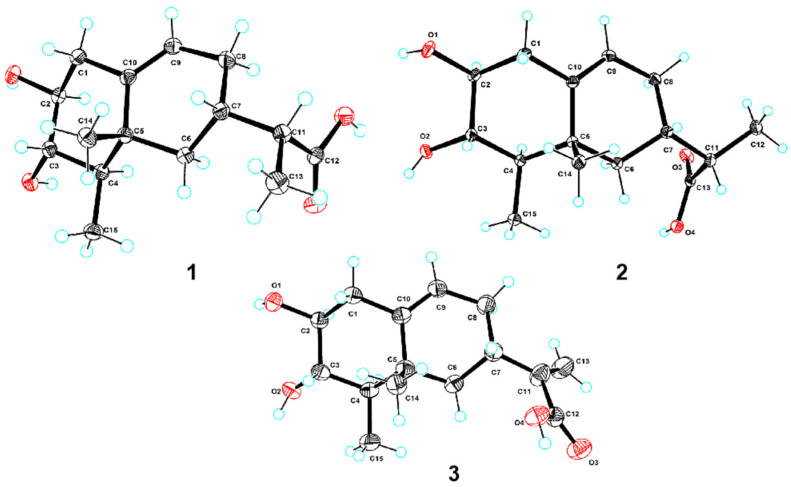
Perspective ORTEP drawing of X-ray structures of **1**–**3**.

Compound **1** was obtained as colorless needles. Its molecular formula was determined to be C_15_H_24_O_4_ based on the high-resolution electrospray ionization mass spectroscopy (HRESIMS) data, corresponding to 4 degrees of unsaturation. Analysis of the ^1^H NMR data (Table 1) revealed the presence of one olefinic proton (*δ*_H_ 5.31 (1H, d, *J* = 5.1 Hz, H-9)), five sp^3^ methines with two of them being attached to oxygen atoms (*δ*_H_ 3.03 (1H, overlapped, H-2), 3.03 (1H, overlapped, H-3), 1.00 (1H, m, H-4), 1.74 (1H, dd, *J* = 7.0, 2.5 Hz, H-7), 2.15 (1H, overlapped, H-11)), three methylenes (*δ*_H_ 2.15 (1H, overlapped, H-1a), 2.06 (1H, overlapped, H-1b), 1.61 (1H, overlapped, H-6a), 0.89 (1H, m, H-6b), 1.61 (1H, overlapped, H-8a), 1.92 (1H, m, H-8b)) and three methyls (*δ*_H_ 1.01 (3H, d, *J* = 7.0 Hz, H_3_-13), 0.88 (3H, s, H_3_-14), 0.86 (3H, d, *J* = 6.7 Hz, H_3_-15)). In addition to the aforementioned 12 proton linked carbon signals, the ^13^C NMR and HSQC data also showed three additional carbon signals, including one carboxylic carbonyl (*δ*_C_ 177.0 (C-12)), one olefinic carbon (*δ*_C_ 141.0 (C-10)) and one quaternary carbon (*δ*_C_ 38.0 (C-5)). The NMR data were similar to those of the sesquiterpenoid, artefreynic acid A [16]. Combined with the two spin systems H_2_-1/H-2/H-3/H-4/H_3_-15 and H_2_-6/H-7/H_2_-8/H-9 observed in the ^1^H-^1^H COSY spectrum, as well as the HMBC correlations of H_2_-1/C-5, C-9, C-10 and H_3_-14/C-4, C-5, C-6 (Figure 2A), **1** was shown to be a sesquiterpenoid derivative with a 6/6 bicyclic skeleton, with the two methyl groups attached to C-4 and C-5, respectively. The HMBC correlation of H-7, H_3_-13/C-12 and ^1^H-^1^H COSY correlation of H-7/H-11/H_3_-13 confirmed the presence of a branched carboxylic acid moiety attached at C-7. Considering the chemical shifts of CH-2 (*δ*_H/C_ 3.03/74.7) and CH-3 (*δ*_H/C_ 3.03/75.5) as well as the molecular formula, it was deduced that the two hydroxyl groups were attached to C-2 and C-3, respectively. Based on these data, the planar structure of **1** was established as shown in Figure 1. The relative configuration of the rings in **1** was presumed to be rel-(2*S*, 3*S*, 4*R*, 5*R*, 7*S*) based on the NOESY correlations of H-2/H-4, H_3_-15/H-3, H_3_-15/H_3_-14 and H_3_-14/H-7 (Figure 2B). However, the peaks of H-2 and H-3 overlapped heavily and the configuration of the CH-11 side chain could not be unambiguously determined. Finally, the absolute configuration of **1** was established as 2*S*, 3*S*, 4*R*, 5*R*, 7*S*, 13*R* by X-ray diffraction analysis (Figure 3), and the compound was named talaroterpene A (**1**). 

Compound **2** was obtained as colorless needles and its molecular formula was determined as C_15_H_24_O_4_ by HRESIMS data, indicating the same degree of unsaturation as **1**. Analysis of the 1D NMR data (Table 1) showed that **2** was similar to **1**. Careful analysis of the 2D NMR data of **2** and **1** (Figure 2) revealed that they were diastereomers with the same planar structure. Based on the NOESY correlations of H-2/H-4, H_3_-15/H-3, H_3_-15/H_3_-14 and H_3_-14/H-7, the relative configuration of **2** was presumed to be rel-(2*S*, 3*S*, 4*R*, 5*R*, 7*S*), which was the same as that of **1**, indicating that the difference might lie in the configuration of the CH-11 side chain. The absolute configuration of **2** was finally established as 2*S*, 3*S*, 4*R*, 5*R*, 7*S*, 13*S* by X-ray diffraction analysis (Figure 3), and **2** was named talaroterpene B.

Compound **3** was obtained as colorless crystals. Its molecular formula was determined as C_15_H_22_O_4_ by HRESIMS data, indicating 5 degrees of unsaturation, which was one higher than that of **1**/**2**. Analysis of the 1D NMR data of **3** (Table 2) revealed similarities with **1**/**2**, except for the presence of two additional olefinic carbon–hydrogen signals (*δ*_H/C_ 6.04, 5.51/103.8; *δ*_C_ 146.2) and the absence of one methyl and one methine signal, suggesting that the -CH-CH_3_ moiety of the side chain had been oxidized to a double bond. The HMBC correlations of H_2_-13/C-7, 11, 12 (Figure 2A) of **3** confirmed this hypothesis. Based on the NOESY correlations of H-2/H-4, H_3_-15/H-3, H_3_-15/H_3_-14 and H_3_-14/H-7, the relative configuration of **3** was presumed to be rel-(2*S*, 3*S*, 4*R*, 5*R*, 7*S*) which was the same as that of **1**/**2**, suggesting that they shared the same relative configuration. Finally, the absolute configuration of **3** was confirmed as 2*S*, 3*S*, 4*R*, 5*R*, 7*S* by X-ray diffraction analysis, and **3** was named talaroterpene C.

**Figure 4:**
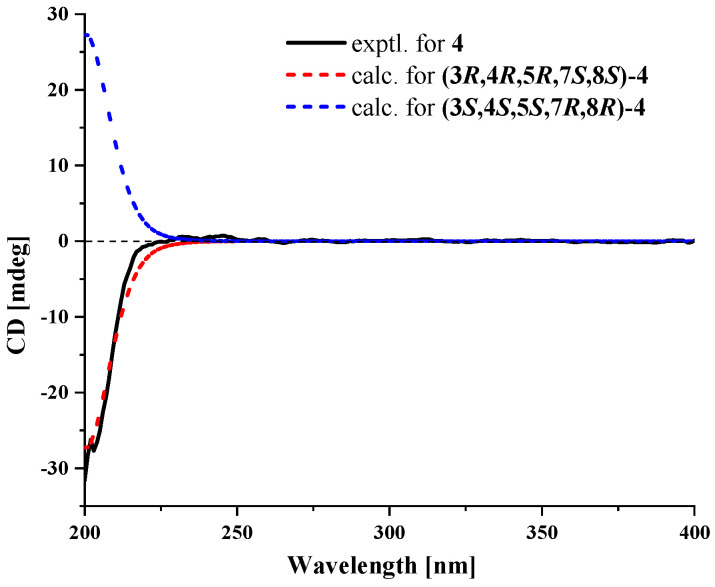
Experimental and calculated ECD spectra of **4**.

Compound **4** was obtained as a colorless oil, and its molecular formula was found to be two oxygen atoms less than that of **3**. Analysis of the NMR data (Table 2) showed that **4** was similar to **3**, except for the appreciable differences in the presence of CH_2_-2, CH-8, CH_2_-12 and a tetrahydrofuran ring in **4** versus the presence of CH-2, CH_2_-8 and CO-12 in **3**. The ^1^H-^1^H COSY correlations of H_2_-1/H_2_-2/H-3/H-4/H_3_-15 indicated that **4** was one hydroxy group less at C-2 compared to **3**. The HMBC correlations (Figure 2A) of H_2_-12/C-7, C-8, C-11 and H_2_-13/C-7, C-11, C-12 of **4** confirmed the formation of a tetrahydrofuran ring. The relative configuration of the rings in **4** was determined to be rel-(3*R*, 4*R*, 5*R*, 7*S,* 8*S*) based on the NOESY correlations (Figure 2B) of H_3_-15/H-3, H_3_-15/H_3_-14, H_3_-14/H-7, H_3_-14/H-8 and H-7/H-8. Based on the calculated electronic circular dichroism (ECD) for the 3*R*, 4*R*, 5*R*, 7*S*, 8*S* and 3*S*, 4*S*, 5*S*, 7*R*, 8*R* configuration (Figure 4), the experimental ECD spectrum of 4 showed a good match with the calculated one with the 3*R*, 4*R*, 5*R*, 7*S*, 8*S* configuration. Thus, **4** was named talaroterpene D.

### 2.2. Bioactivity Assay

**Figure 5:**
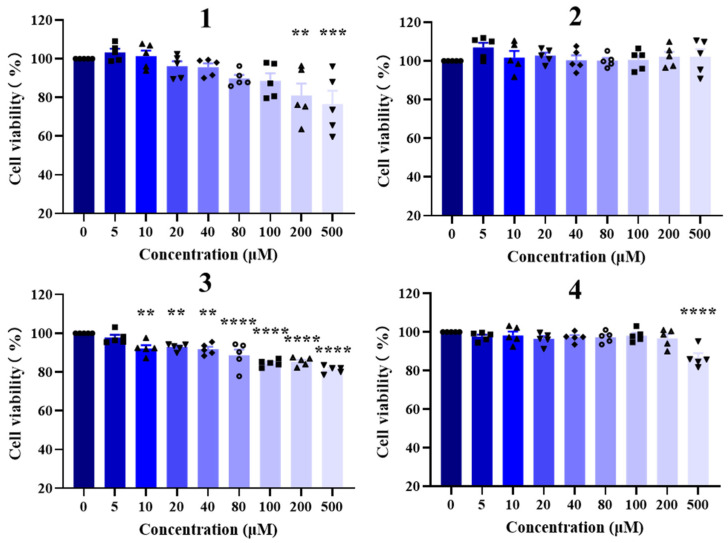
The cytotoxic effects of **1**–**4** against HepG2 cells. ** *p* < 0.01; *** *p* < 0.001; **** *p* < 0.0001.

For this work, compounds **1**–**4** were evaluated with their regulatory activities on nuclear receptors in HepG2 cells [10]. Firstly, CCK8 assay was taken to evaluate the toxicity against HepG2 cells (Figure 5). Compounds **2** and **4** are non-toxic to cells at concentrations of 200 μM and below. Compounds **1** and **3** showed weak cytotoxic effects on cells at concentration of 200 μM, with IC_50_ > 500 μM.

**Figure 6:**
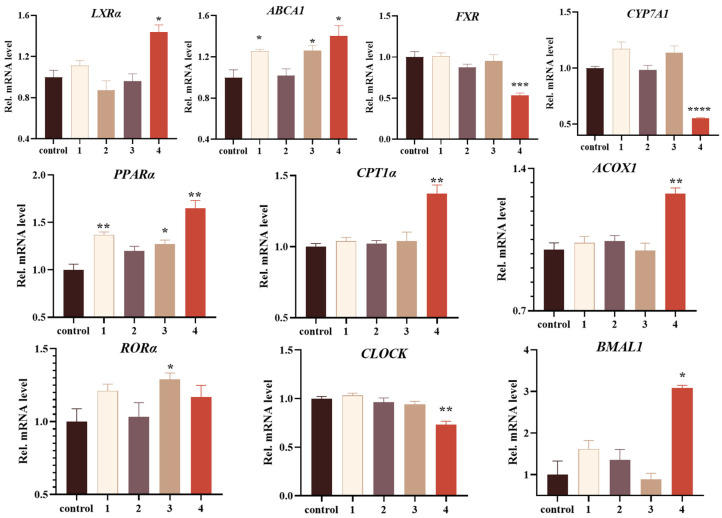
The regulatory activities of **1**–**4** (200 μM) on nuclear receptors in HepG2 cells. The results were mean ± SEM (*n* = 3, * *p* < 0.05, ** *p* < 0.01, *** *p* < 0.001 and **** *p* < 0.0001 compared to the control group).

It was observed that talaroterpene D (**4**), with a non-toxic concentration of 200 μM, notably stimulated *LXRα* and its downstream target gene *ABCA1*, and significantly suppressed the expression of *FXR* and downstream *CYP7A1*. Compounds **1** and **3** (200 μM) could also stimulate expression of *ABCA1*, but there were no obvious activities with respect to *LXRα*, *FXR* and *CYP7A1*. Additionally, compounds **1**, **3** and **4** (200 μM) exhibited varying degrees of activation on *PPARα*. Talaroterpene D (**4**) also showed activation on downstream target genes *CPT1α* and *ACOX1*. Furthermore, talaroterpene D (**4**) showed minimal impact on *RORα* itself; however, it induced significant alterations in the expression of downstream target genes *CLOCK* and *BMAL1* of *RORα*, with a concentration of 200 μM, possibly due to direct binding interactions with RORα (Figure 6). 

**Figure 7:**
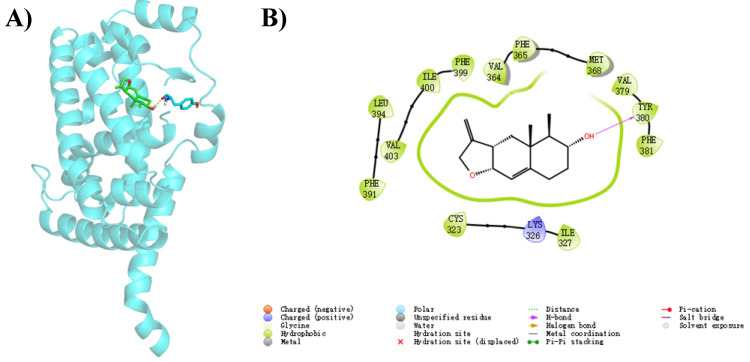
The docking results of **4** with RORα. (**A**) Three-dimensional and (**B**) two-dimensional binding mode of **4** with RORα (PDB code: 1N83) predicted by in silico molecular docking.

To further elucidate the binding mode of compounds **1**–**4** with RORα, a homology model of RORα (PDB code: 1N83) was selected for in silico molecular docking analysis [17]. The docking results demonstrated that compound **4** fits comfortably within the binding pocket of RORα (Figure 7A), yielding a binding score of −8.613. The 2D binding models of **4** with RORα (Figure 7B) revealed that the hydroxy group forms hydrogen bonds with the active-site residue TYR380. Similarly, the hydroxyl groups of compounds **1**–**3** form hydrogen bond interactions with TYR380 in the RORα (Figures S41–S43). The above results indicate that the presence of the hydroxyl group is beneficial for enhancing the binding interactions between the compounds and RORα. Compounds **1**–**3** all contain a carboxyl group, which under normal physiological pH conditions, typically exists in an ionized form, leading to a decrease in lipophilicity, thus hindering drug absorption and ultimately resulting in reduced biological activity, suggesting that the presence of the carboxyl group is not favorable for enhancing the RORα activity. Additionally, **3** experiences spatial conflict between the alkene group on the side chain and the RORα active site, leading to a reduction in its interactions with RORα. In contrast, compound **4**, which also contains an alkene group, has a larger gap between the cyclic alkene and the RORα active site due to the cyclization, thereby eliminating the spatial conflict and enhancing its interactions with RORα. For future structural optimization and modification, it is promising to explore the possibility of cyclizing the carboxyl-containing side chains, which may further improve the biological activity of talaroterpenes.

## 3. Materials and Methods

### 3.1. General Experimental Procedures

Optical rotations were determined using an Anton Paar MPC 500 polarimeter (Anton, Graz, Austria). UV and IR spectra were recorded using a Shimadzu UV-2600 PC spectrometer (Shimadzu, Beijing, China) and an IR Affinity-1 spectrometer (Shimadzu), respectively. A Quantum-I Plus 500 Hz NMR spectrometer (Q-one Instrument Co., Ltd., Wuhan, China) operating at 500 MHz for ^1^H and 125 MHz for ^13^C was used. HRESIMS were acquired on a Bruker maXis Q-TOF mass spectrometer (Bruker BioSpin International AG, Fällanden, Switzerland). HPLC was performed on the Hitachi Primaide (Hitachi, Tokyo, Japan) with a DAD detector, using an ODS column (YMC-pack ODS-A, 10 × 250 mm, 5 μm). X-ray diffraction was performed on an XtalLAB PRO diffractometer (Rigaku, Akishima-shi, Japan) with Cu Kα radiation.

### 3.2. Fungal Material

A fungal strain identified as *Talaromyces* sp. SCSIO 41412 was isolated from a sediment sample obtained from the Gaoqiao Mangrove in Zhanjiang City, Guangdong Province, China, in August 2021. The taxonomic assignment of this fungus was based on analysis of the internally transcribed spacer (ITS) region of the ribosomal DNA (rDNA), and the ITS sequence has been deposited in GenBank under accession number PP001498. The fungal strain was stored on malt extract agar (15 g malt extract, 18 g agar, 10 g sea salt and 1 L water) at 4 °C, and deposited in the CAS Key Laboratory of Tropical Marine Bioresources and Ecology, South China Sea Institute of Oceanology, Chinese Academy of Sciences, Guangzhou, China.

### 3.3. Fermentation, Extraction and Isolation

The fungal strain *Talaromyces* sp. SCSIO 41412 was cultured in 200 mL of seed medium (15 g malt extract, 10 g sea salt and 1 L water) on a rotary shaker (180 rpm) at 28 °C for 3 days. This seed culture was then used to inoculate a large-scale fermentation, which was incubated statically at 26 °C for 28 days using a rice medium (200 g rice, 2% sea salt and 230 mL water) in 47 Erlenmeyer flasks. The entire fermented culture was extracted with ethyl acetate (EtOAc) three times, yielding a total extract of 326.1 g. The EtOAc extract was chromatographed over a silica gel column eluted with CH_2_Cl_2_/petroleum ether (0:1, 1:1, 1:0) and CH_3_OH/CH_2_Cl_2_ (1:99, 2:98, 3:97, 5:95, 10:90, 20:80, 50:50) to yield ten fractions (Frs.1–10). Fr.4 was subjected to ODS silica gel chromatography and eluted with CH_3_OH/H_2_O (5–100%) to yield 14 subfractions. Fr.4-3 was further separated by semi-preparative HPLC (50% CH_3_CN/H_2_O, 3 mL/min, YMC-pack ODS-A, 10 × 250 mm, 5 μm) to afford Fr.4-3-3, which was then purified by HPLC (68% CH_3_OH/H_2_O, 2.5 mL/min) to yield **4** (6.8 mg, *t*_R_ = 15.5 min). Fr.6 was subjected to ODS silica gel chromatography and eluted with CH_3_OH/H_2_O (5–100%) to yield 10 subfractions. Fr.6-4 was separated by semi-preparative HPLC (33% CH_3_CN/H_2_O, 3.0 mL/min, YMC-pack ODS-A, 10 × 250 mm, 5 μm) to afford Fr.6-4-1, which was further separated by semi-preparative HPLC (50% CH_3_CN/H_2_O, 3.0 mL/min, YMC-pack ODS-A, 10 × 250 mm, 5 μm) to yield **2** (9.9 mg, *t*_R_ = 16.0 min), **1** (119.8 mg, *t*_R_ = 16.2 min) and **3** (49.1 mg, *t*_R_ = 16.9 min).

### 3.4. Spectroscopic Data of Compounds

Talaroterpene A (**1**): colorless needles; ${\left[\alpha \right]}_{\;D}^{25}$ +14.5 (*c* 0.1, CH_3_OH); ECD (0.3 mg/mL, CH_3_OH) *λ*_max_ (Δ*ε*) 224 (−2.36); UV (CH_3_OH) *λ*_max_ (log *ε*) 200 (3.88), 320 (2.73) nm; IR *ν*_max_ 3398, 1697, 1558, 1541, 1456, 1047 cm^−1^; ^1^H and ^13^C NMR, Table 1; HRESIMS *m*/*z* 286.2014 [M + NH_4_]^+^ (calcd for C_15_H_28_NO_4_^+^, 286.2013).

Talaroterpene B (**2**): colorless needles; ${\left[\alpha \right]}_{\;D}^{25}$ +18.8 (*c* 0.1, CH_3_OH); ECD (0.3 mg/mL, CH_3_OH) *λ*_max_ (Δ*ε*) 202 (−2.35); UV (CH_3_OH) *λ*_max_ (log *ε*) 200 (3.81) nm; IR *ν*_max_ 3375, 2968, 2914, 1703, 1550, 1448, 1373, 1251, 1199, 1041, 1024 cm^−1^; ^1^H and ^13^C NMR, Table 1; HRESIMS *m*/*z* 286.2018 [M + NH_4_]^+^ (calcd for C_15_H_28_NO_4_^+^, 286.2013).

Talar terpene C (**3**): colorless block crystal; ${\left[\alpha \right]}_{\;D}^{25}$ +23.5 (*c* 0.1, CH_3_OH); ECD (0.3 mg/mL, CH_3_OH) *λ*_max_ (Δ*ε*) 224 (−2.94); UV (CH_3_OH) *λ*_max_ (log *ε*) 200 (3.89) nm; IR *ν*_max_ 3338, 1681, 1541, 1373, 1253, 1041 cm^−1^; ^1^H and ^13^C NMR, Table 2; HRESIMS *m*/*z* 265.1451 [M-H]^−^ (calcd for C_15_H_21_O_4_^−^, 265.1445).

Talaroterpene D (**4**): colorless oil; ${\left[\alpha \right]}_{\;D}^{25}$ +3.0 (*c* 0.1, CH_3_OH); ECD (0.3 mg/mL, CH_3_OH) *λ*_max_ (Δ*ε*) 203 (−27.70); UV (CH_3_OH) *λ*_max_ (log *ε*) 200 (3.90) nm; IR *ν*_max_ 3392, 1653, 1373, 1026 cm^−1^; ^1^H and ^13^C NMR, Table 2; HRESIMS *m*/*z* 235.1694 [M + H]^+^ (calcd for C_15_H_23_O_2_^+^, 235.1693).

### 3.5. X-ray Crystallographic Analysis

The X-ray diffraction data for **1**–**3** were collected using an XtaLAB PRO diffractometer with Cu K*α* radiation, with the crystals grown from methanol by slow evaporation. The crystal structures were solved using SHELXS97, expanded through difference Fourier techniques, and then refined by full-matrix least-squares methods. All non-hydrogen atoms were refined anisotropically, and the hydrogen atoms were fixed at calculated positions. The crystallographic data for **1**–**3** have been deposited in the Cambridge Crystallographic Data Centre.

Crystal Data for Talaroterpene A (**1**): C_15_H_24_O_4_, *M* = 268.34, monoclinic, space group *P*2_1_, *a* = 10.23266 (8) Å, *b* = 6.72249 (4) Å, *c* = 11.56363(9) Å, *β* = 115.5824(10)°, *V* = 717.467 (11) Å^3^, *Z* = 2, *T* = 100.00 (10) K, *μ*(Cu K*α*) = 0.719 mm^−1^, *D*_calc_ = 1.242 g/cm^3^, 13,788 reflections measured (8.478° ≤ 2*Θ* ≤ 148.13°), 2843 unique (*R*_int_ = 0.0420, *R*_sigma_ = 0.0236) which were used in all calculations. The final *R*_1_ was 0.0280 (*I* > 2*σ*(*I*)) and *wR*_2_ was 0.0724. The Flack parameter was 0.06 (7) (CCDC 2372513).

Crystal Data for Talaroterpene B (**2**): C_15_H_24_O_4_, *M* = 268.34, monoclinic, space group *P*2_1_, *a* = 9.09200 (10) Å, *b* = 7.93080 (10) Å, *c* = 9.95020 (10) Å, *β* = 96.4630 (10)°, *V* = 712.918 (14) Å^3^, *Z* = 2, *T* = 100.00 (10) K, *μ* (Cu K*α*) = 0.724 mm^−1^, *D*_calc_ = 1.250 g/cm^3^, 10,853 reflections measured (8.944° ≤ 2*Θ* ≤ 148.192°), 2834 unique (*R*_int_ = 0.0223, *R*_sigma_ = 0.0158) which were used in all calculations. The final *R*_1_ was 0.0289 (*I* > 2*σ*(*I*)) and *wR*_2_ was 0.0787. The Flack parameter was 0.09 (5) (CDCC 2372514).

Crystal Data for Talaroterpene C (**3**): C_15_H_22_O_4_, *M* = 266.32, orthorhombic, space group *P*2_1_2_1_2_1_, *a* = 7.24180 (10) Å, *b* = 11.42500 (10) Å, *c* = 16.6083 (2) Å, *V* = 1374.13 (3) Å^3^, *Z* = 4, *T* = 99.99 (10) K, *μ*(Cu K*α*) = 0.751 mm^−1^, *D*_calc_ = 1.287 g/cm^3^, 12,963 reflections measured (9.396° ≤ 2*Θ* ≤ 148.8°), 2750 unique (*R*_int_ = 0.0569, *R*_sigma_ = 0.0327) which were used in all calculations. The final *R*_1_ was 0.0433 (*I* > 2*σ*(*I*)) and *wR*_2_ was 0.1198. The Flack parameter was 0.09 (13) (CDCC 2372529).

### 3.6. ECD Computation Section

Compound **4** was subjected to conformational searching in Spartan’14 (v1.1.4, Wavefunction, Irvine, CA, USA) using the Molecular Merck force field. The stable conformers of 99% (the relative energy within 2 kcal/mol) were then optimized in methanol solvent at the B3LYP/6-31G (d) level of theory using Gaussian 09 (D.01, Pittsburgh, PA, USA). The optimized low-energy conformations were further analyzed using TDDFT with a polarizable continuum model at the B3LYP/6-311G (d, p) level [18]. The calculated ECD spectra were generated from GaussView 6.0 and Origin 2021, with a half-bandwidth of 0.3 eV, wavelength-corrected by the calculated UV curve, and Boltzmann-weighted to obtain the final calculated ECD spectra.

### 3.7. Cell Culture

HepG2 cells were purchased from the Shanghai Cell Bank, Chinese Academy of Sciences, and cultured in Dulbecco’s modified Eagle’s medium (DMEM, Gibco, New York, NY, USA) supplemented with 10% fetal bovine serum (FBS, ExCell Bio, Suzhou, China) at 37 °C under 5% CO_2_. Mother solutions of 20 mM **1**–**4** were dissolved in dimethyl sulfoxide (DMSO, Tianjin Fuyu Chemical and Industry Factory, Tianjin, China) and then diluted to 200 μM with the cell-culture medium. The control group was treated with a medium containing 1‰ DMSO.

### 3.8. Cytotoxic Bioassay

Cell viability was assessed using the CCK-8 assay (Dojindo, Kumamoto, Japan). Initially, cells were plated at a density of 3000 cells/well in 96-well plates and exposed to varying concentrations of compounds or a solvent control. Following a 24 h incubation period, CCK-8 reagent was introduced, and the absorbance of triplicate samples was measured at 450 nm using an Envision 2104 multilabel reader (PerkinElmer, Waltham, MA, USA). Dose-response curves were generated to determine the IC_50_ values with Prism 5.0 software (GraphPad, San Diego, CA, USA).

### 3.9. RT-qPCR

Total RNA was extracted using the RNAprep Kit (RE-03014, FOREGENE, Chengdu, China) and reverse transcribed to cDNA using RT Master Mix (RR037A, Takara, Shiga, Japan). RT-qPCR was performed using SYBR Green Master Mix (A6002, Promega, Madison, WI, USA). The data were normalized to the housekeeping gene GAPDH. Primers used are listed in Table S1.

### 3.10. Statistical Analysis

Prism 9 software (GraphPad, San Diego, CA, USA) was used to perform statistical tests. Groups (*n* = 6) were compared by one-way analysis of variance (ANOVA). Nonparametric data were log-transformed for statistical analysis, or when this failed to normalize the data, Mann–Whitney tests were used. *p* < 0.05 was significant (* *p* < 0.05, ** *p* < 0.01, *** *p* < 0.001, and **** *p* < 0.0001 compared to the control group).

## 4. Conclusions

In summary, this study identified four new sesquiterpenoid compounds, talaroterpenes A–D (**1**–**4**), isolated from the mangrove-derived fungus *Talaromyces* sp. SCSIO 41412. Through detailed structural characterization, the absolute configurations of these compounds were elucidated. Those new sesquiterpenoids were evaluated with their regulatory activities on nuclear receptors in HepG2 cells. Under the non-toxic concentration of 200 μM, **1**, **3** and **4** exhibited varying degrees of activation on *ABCA1* (downstream target of LXRα) and *PPARα*, while **4** showed the strongest activities. Furthermore, **4** induced significant alterations in the expression of downstream target genes *CLOCK* and *BMAL1* of RORα, and the in silico molecular docking analysis supported the direct binding interactions of **4** with RORα protein. This study revealed that the sesquiterpenoid talaroterpene D (**4**), as a new potential non-toxic modulator of nuclear receptors, holds promise as a lead compound for the development of candidate drugs for metabolic or cardiovascular diseases.

## Figures and Tables

**Table 1:** ^1^H (500 MHz) and ^13^C (125 MHz) NMR data of **1** and **2** in DMSO-*d*_6_.

Pos.	1		2	
	*δ*_C_ Type	*δ*_H_ (*J* in Hz)	*δ*_C_ Type	*δ*_H_ (*J* in Hz)
1	39.3, CH_2_	2.15, overlapped 2.06, m	39.3, CH_2_	2.16, overlapped 2.06, m
2	74.7, CH	3.03, overlapped	74.7, CH	3.02, overlapped
3	75.5, CH	3.03, overlapped	75.5, CH	3.02, overlapped
4	47.3, CH	1.00, m	47.4, CH	0.98, m
5	38.0, C		37.9, C	
6	42.3, CH_2_	1.61, overlapped 0.89, m	40.5, CH_2_	1.67, m 0.83, m
7	32.4, CH	1.74, m	32.7, CH	1.72, overlapped
8	28.5, CH_2_	1.92, m 1.61, overlapped	29.7, CH_2_	1.89, m 1.72, overlapped
9	120.1, CH	5.31, d (5.1)	120.2, CH	5.30, d (5.0)
10	141.0, C		140.9, C	
11	44.4, CH	2.15, overlapped	43.8, CH	2.16, overlapped
12	177.0, C		176.9, C	
13	13.9, CH_3_	1.01, d (7.0)	13.9, CH_3_	1.03, d (6.9)
14	19.1, CH_3_	0.88, s	19.1, CH_3_	0.89, overlapped
15	10.8, CH_3_	0.86, d (6.7)	10.8, CH_3_	0.89, overlapped

**Table 2:** ^1^H (500 MHz) and ^13^C (125 MHz) NMR data of **3** and **4** in DMSO-*d*_6_.

Pos.	3		4	
	*δ*_C_ Type	*δ*_H_ (*J* in Hz)	*δ*_C_ Type	*δ*_H_ (*J* in Hz)
1	39.3, CH_2_	2.19, dd (13.3, 4.8) 2.11, d (10.3)	30.4, CH_2_	2.22, m 2.10, dt (14.0, 4.0)
2	74.8, CH	3.04, overlapped	36.0, CH_2_	1.88, m 1.13, m
3	75.5, CH	3.04, overlapped	69.6, CH	3.29, m
4	47.3, CH	1.05, overlapped	49.7, CH	0.96, m
5	38.5, C		38.3, C	
6	43.4, CH_2_	1.70, d (12.5) 1.05, overlapped	40.1, CH_2_	1.65, dd (12.9, 5.0) 1.06, m
7	31.2, CH	2.06, m	38.2, CH	2.59, m
8	31.1, CH_2_	2.64, m 1.81, m	74.6, CH	3.97, m
9	120.3, CH	5.36, d (5.4)	117.3, CH	5.45, s
10	140.9, C		149.1, C	
11	146.2, C		153.7, C	
12	168.4, C		69.6, CH_2_	4.35, d (13.4) 4.10, d (13.4)
13	121.9, CH_2_	6.04, s 5.51, s	103.8, CH_2_	5.00, d (1.25) 4.90, s
14	19.1, CH_3_	0.94, s	17.7, CH_3_	0.90, s
15	10.8, CH_3_	0.89, d (6.7)	11.2, CH_3_	0.93, d (4.7)

## Data Availability

Data are contained within the article.

## Supplementary Materials

The following supporting information can be downloaded at: https://www.mdpi.com/article/10.3390/md22090403/s1, the NMR, HRESIMS, UV, IR, and ECD spectra of **1**–**4**; Primers of RT-Qpcr; The docking results of **1**–**3** with RORα.
