# Supplementary material for: New Sesquiterpenoids from the Mangrove-Derived Fungus Talaromyces sp. as Modulators of Nuclear Receptors
Source: Mar Drugs. 2024 Sep 3;22(9):403. doi: 10.3390/md22090403 (PMC11433618; doi:10.3390/md22090403)
Supplement: Supplementary file 1 [file marinedrugs-22-00403-s001.zip › marinedrugs-3155077-supplementary.pdf]

Supporting Information

New Sesquiterpenoids from the Mangrove-Derived Fungus *Talaromyces* sp. as Modulators of Nuclear Receptors

Tanwei Gu [1,†], Jian Cai [2,3,†], Danni Xie [1], Jianglian She [2,3], Yonghong Liu [2], Xuefeng Zhou [2,*] and Lan Tang [1,*]

[1] NMPA Key Laboratory for Research and Evaluation of Drug Metabolism, Guangdong Provincial Key Laboratory of New Drug Screening, School of Pharmaceutical Sciences, Southern Medical University, Guangzhou 510515, China; nanyigtw@163.com (T.G.); hc2000822@163.com (D.X.)

[2] CAS Key Laboratory of Tropical Marine Bio-resources and Ecology/Guangdong Key Laboratory of Marine Materia Medica, South China Sea Institute of Oceanology, Chinese Academy of Sciences, Guangzhou 510301, China; caijian19@mails.ucas.ac.cn (J.C.); shejianglian20@mails.ucas.ac.cn (J.S.); yonghongliu@scsio.ac.cn (Y.L.)

[3] University of Chinese Academy of Sciences, Beijing 100049, China;

* Correspondence: xfzhou@scsio.ac.cn (X.Z.); tl405@smu.edu.cn (L.T.)

† These authors contributed equally to this work.

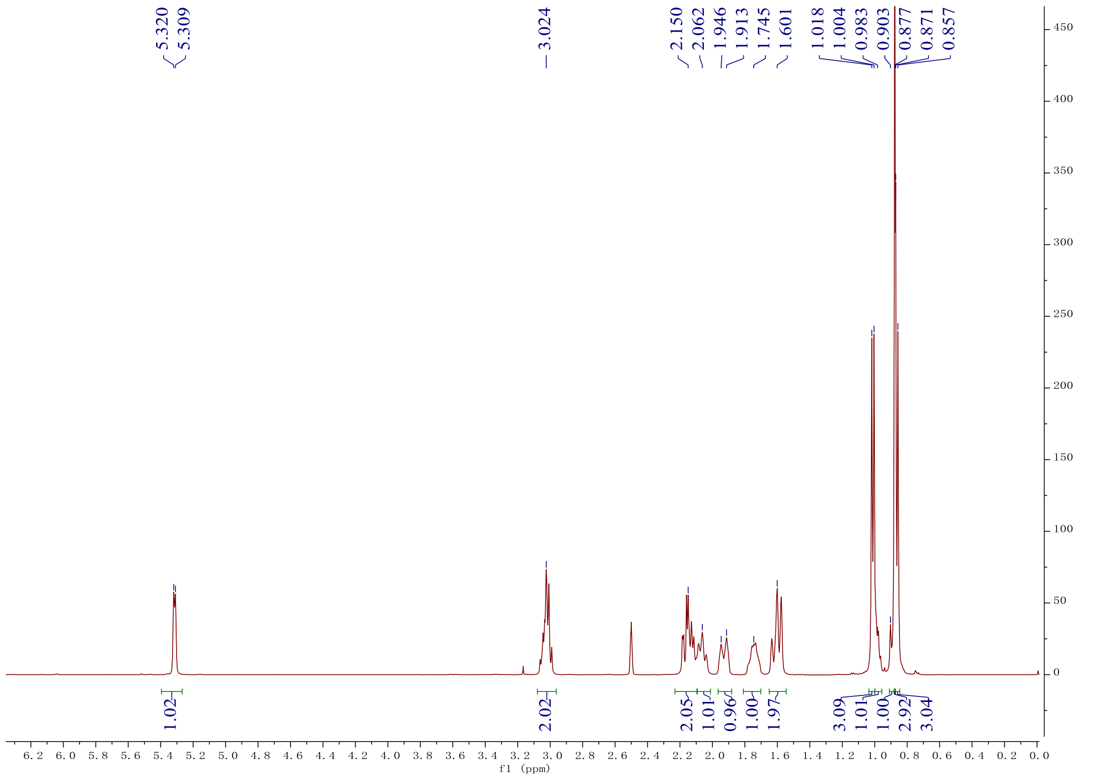

Figure S1. ^{1}H NMR (500 MHz) spectrum of **1** in DMSO-d_6.

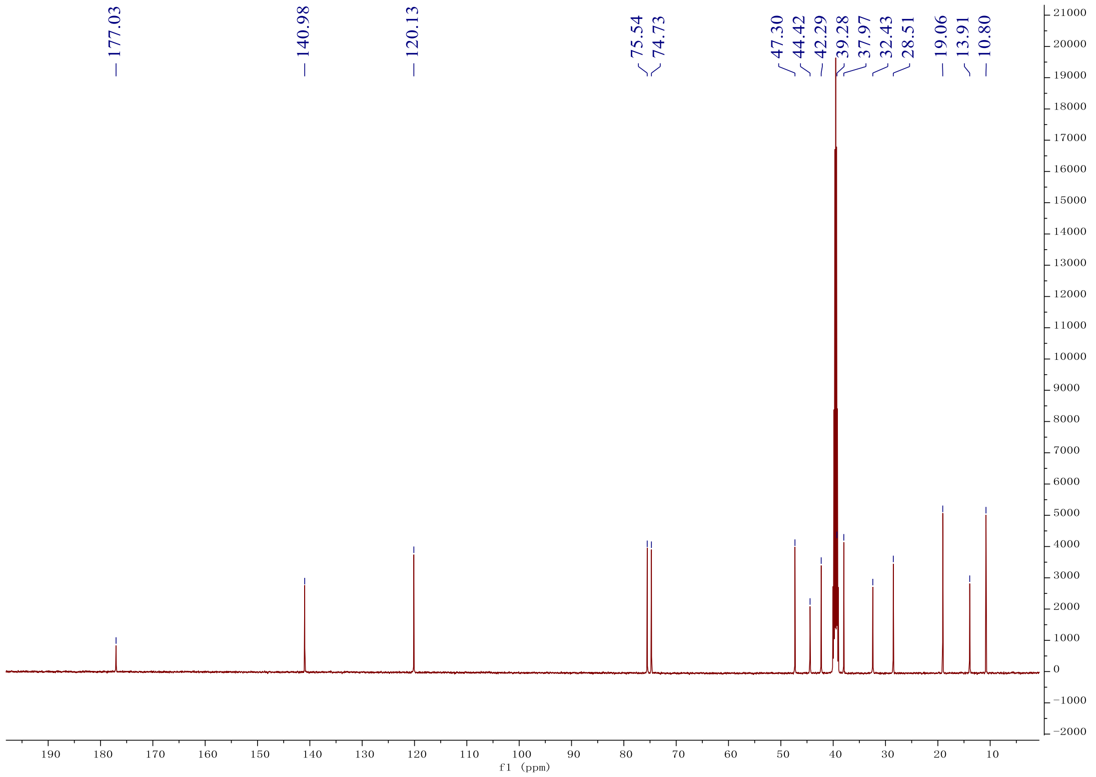

Figure S2. ^{13}C NMR (125 MHz) spectrum of **1** in DMSO-d_6.

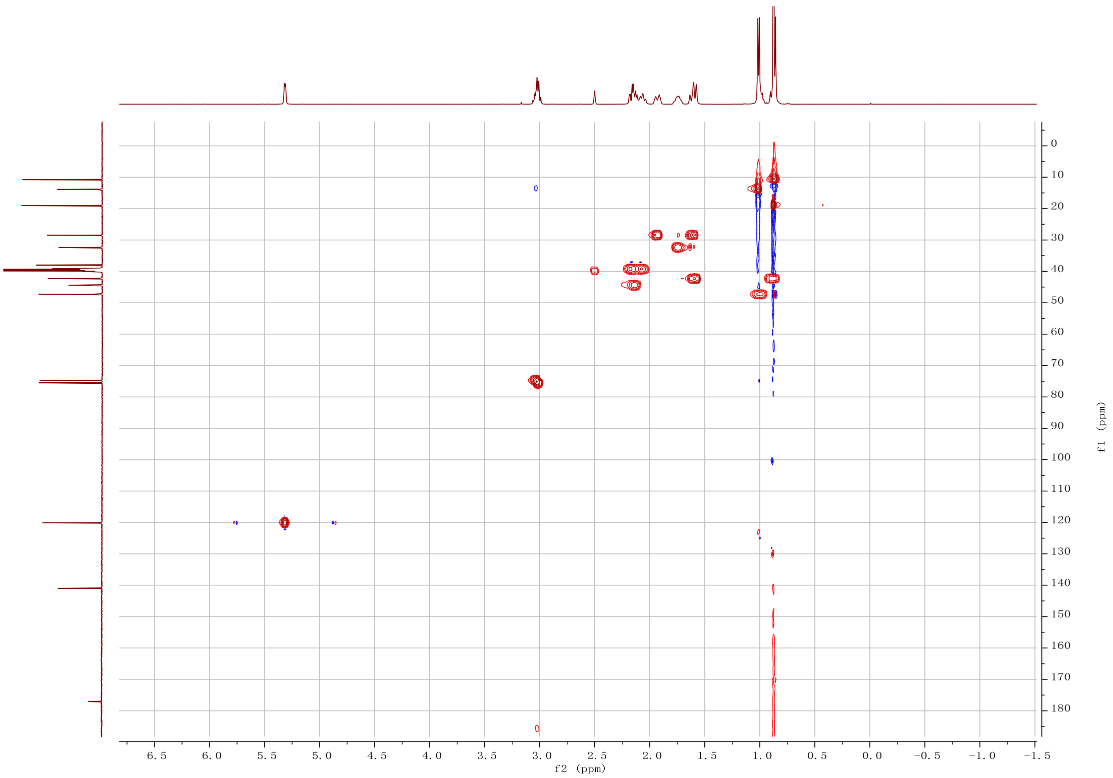

Figure S3. HSQC spectrum of **1** in DMSO-d_6.

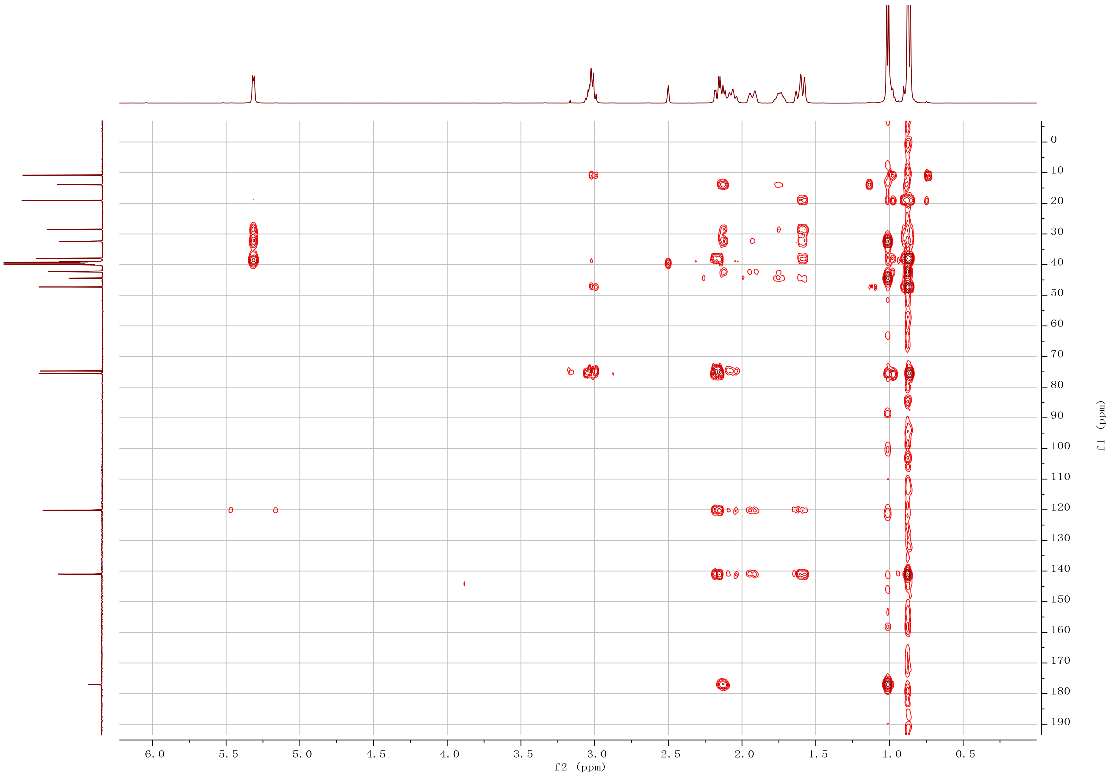

Figure S4. HMBC spectrum of **1** in DMSO-d_6.

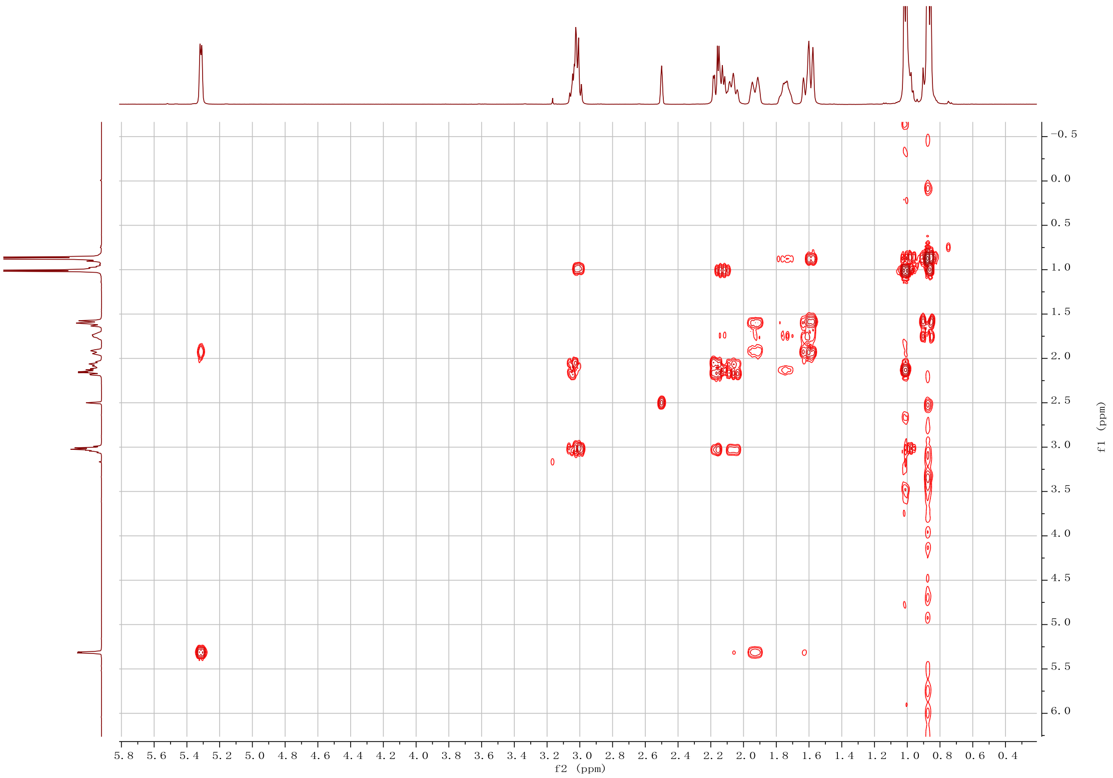

Figure S5. ^{1}H–^{1}H COSY spectrum of **1** in DMSO-d_6.

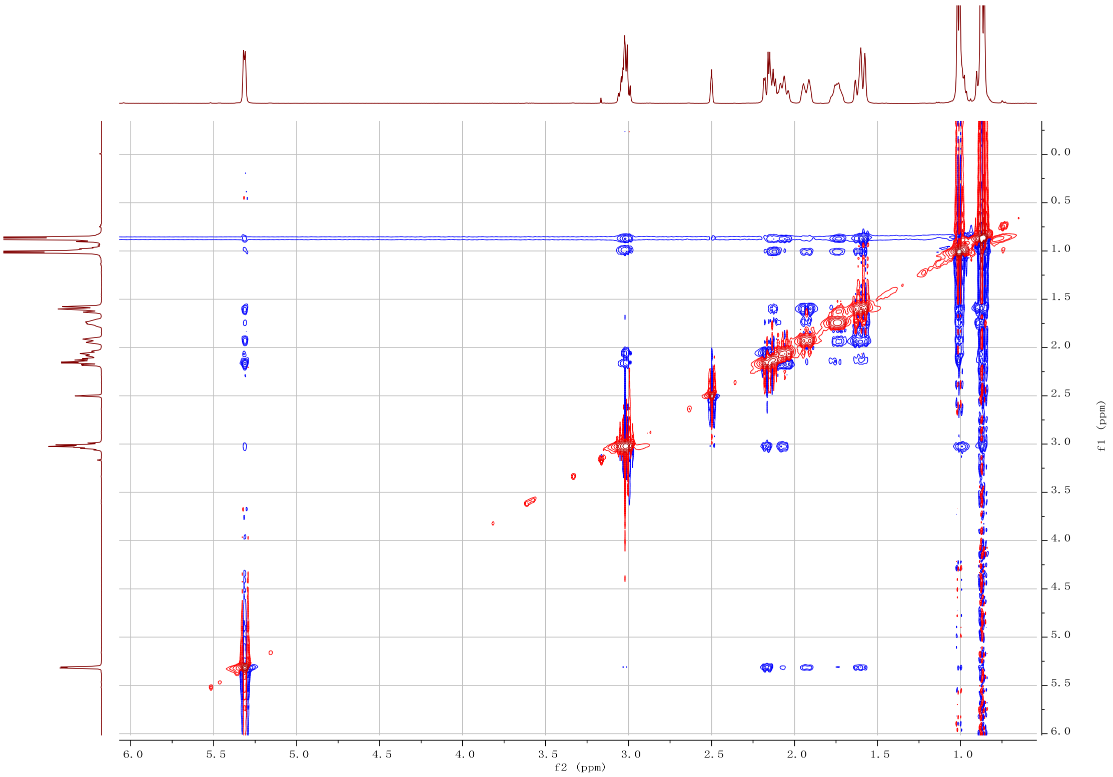

Figure S6. NOESY spectrum of **1** in DMSO-d_6.

Mass Spectrum SmartFormula Report

Analysis Info

Analysis Name	D:\Data\MS\data\202212\caijian_CJC55_pos_31_01_14116.d	Acquisition Date	12/12/2022 11:26:17 AM
Method	LC_Direct Infusion_pos_70-500mz.m	Operator	SCSIO
Sample Name	caijian_CJC 55_pos	Instrument	maXis 255552.00029
Comment			

Acquisition Parameter

Source Type	ESI	Ion Polarity	Positive	Set Nebulizer	0.4 Bar
Focus	Active	Set Capillary	4500 V	Set Dry Heater	180 °C
Scan Begin	100 m/z	Set End Plate Offset	-500 V	Set Dry Gas	4.0 l/min
Scan End	1500 m/z	Set Charging Voltage	0 V	Set Divert Valve	Waste
		Set Corona	0 nA	Set APCI Heater	0 °C

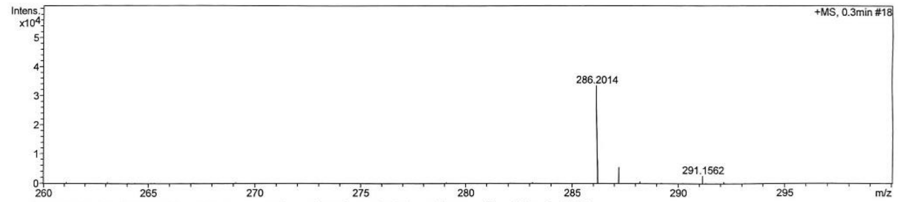

Meas. m/z	#	Ion Formula	Score	m/z	err [ppm]	err [mDa]	mSigma	rdb	e⁻ Conf	N-Rule
286.2014	1	C15H28NO4	100.00	286.2013	0.3	0.1	5.6	2.5	even	ok
291.1562	1	C15H24NaO4	100.00	291.1567	-1.6	-0.5	13.6	3.5	even	ok
537.3434	1	C30H49O8	100.00	537.3422	-2.3	-1.2	179.6	6.5	even	ok
554.3678	1	C30H52NO8	100.00	554.3687	-1.6	-0.9	13.8	5.5	even	ok
559.3226	1	C30H48NaO8	100.00	559.3241	-2.7	-1.5	22.4	6.5	even	ok

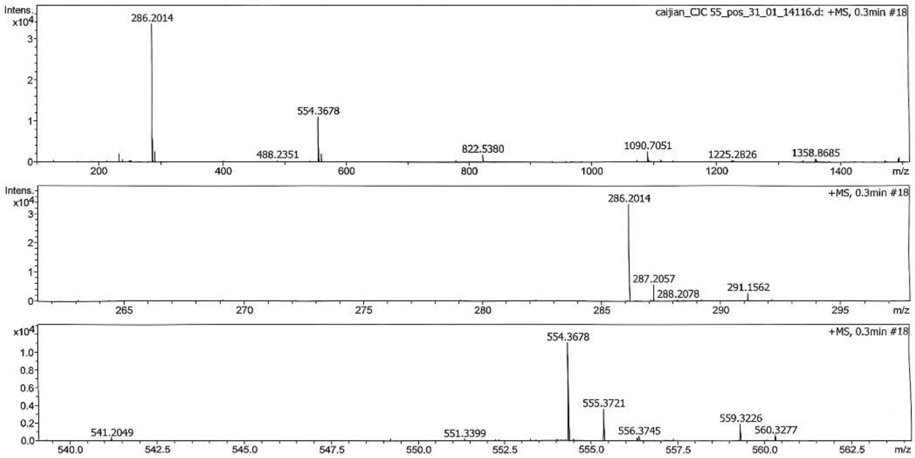

Figure S7. HRESIMS spectrum of **1**.

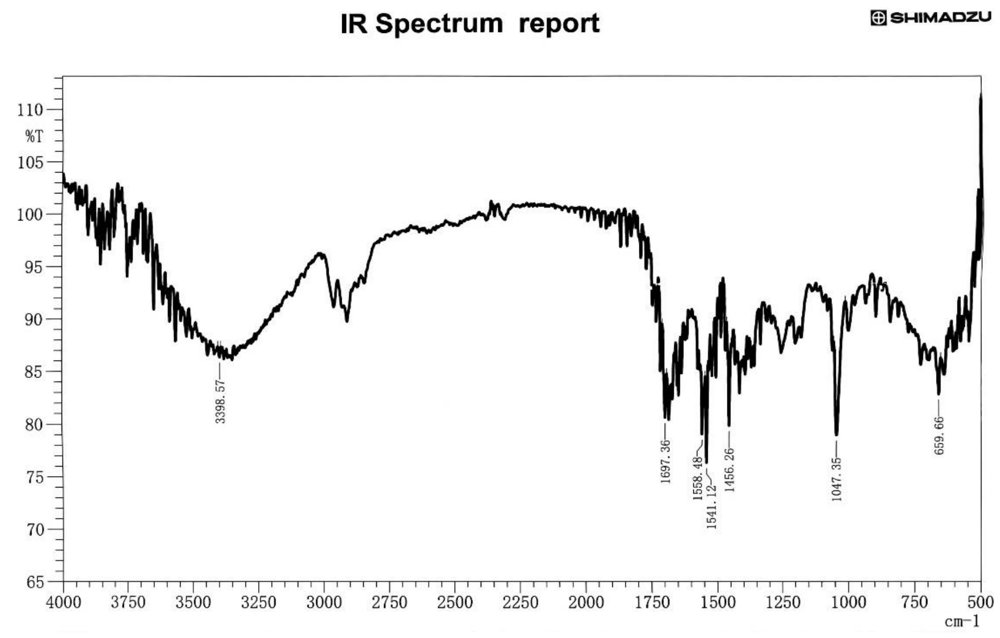

Figure S8. IR spectrum of **1**.

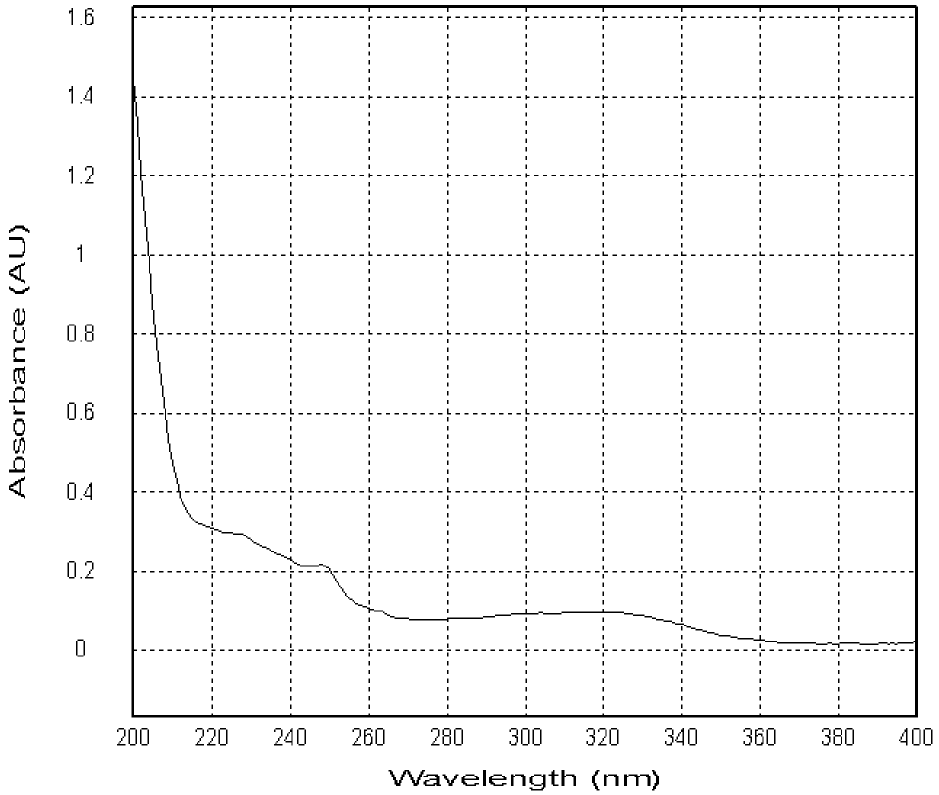

Figure S9. UV spectrum of **1** in MeOH.

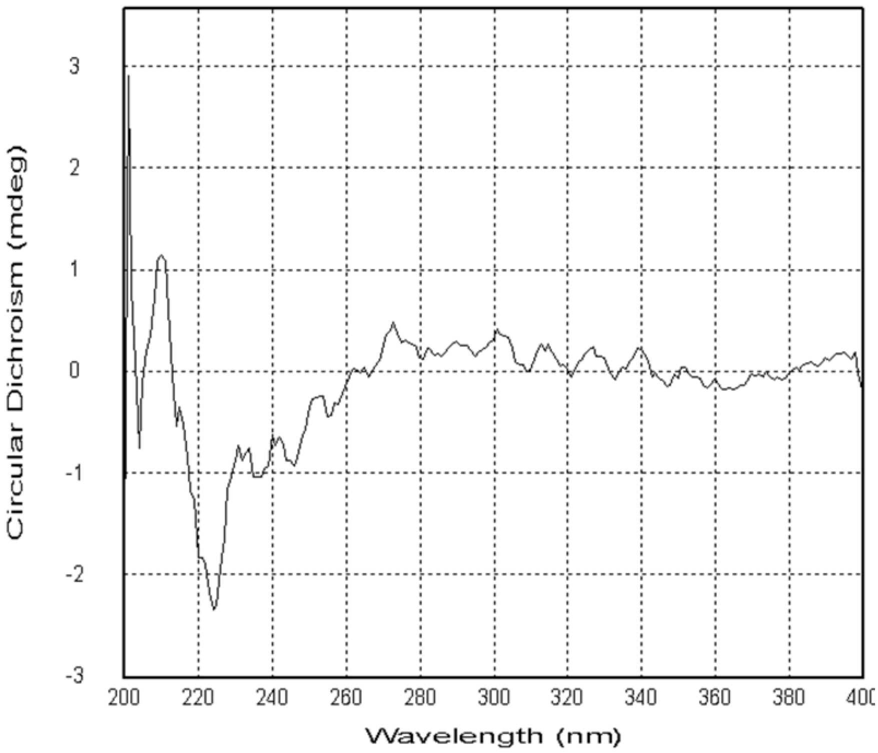

Figure S10. ECD spectrum of **1** in MeOH.

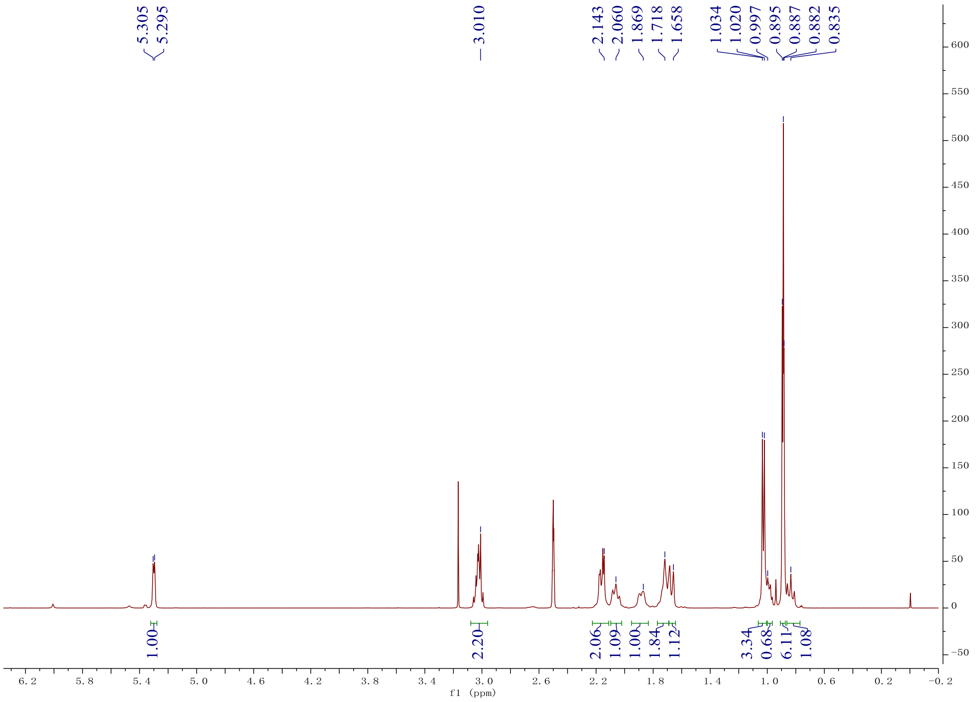

Figure S11. ^{1}H NMR (500 MHz) spectrum of **2** in DMSO-d_6.

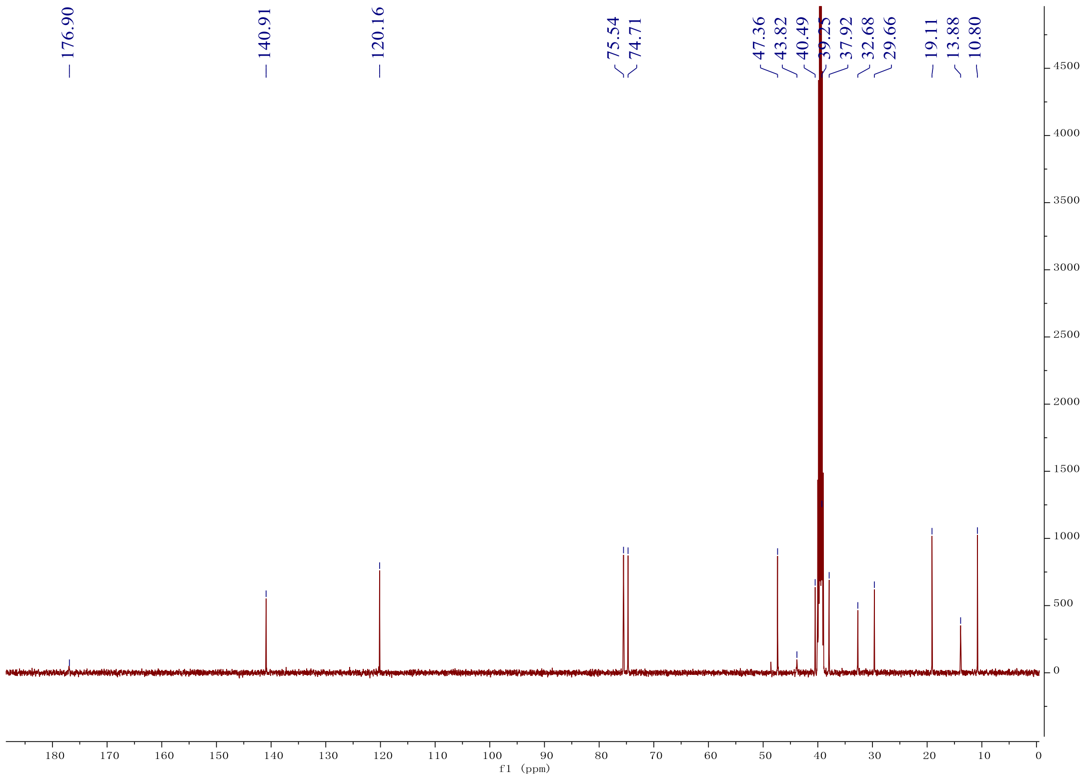

Figure S12. ^{13}C NMR (125 MHz) spectrum of **2** in DMSO-d_6.

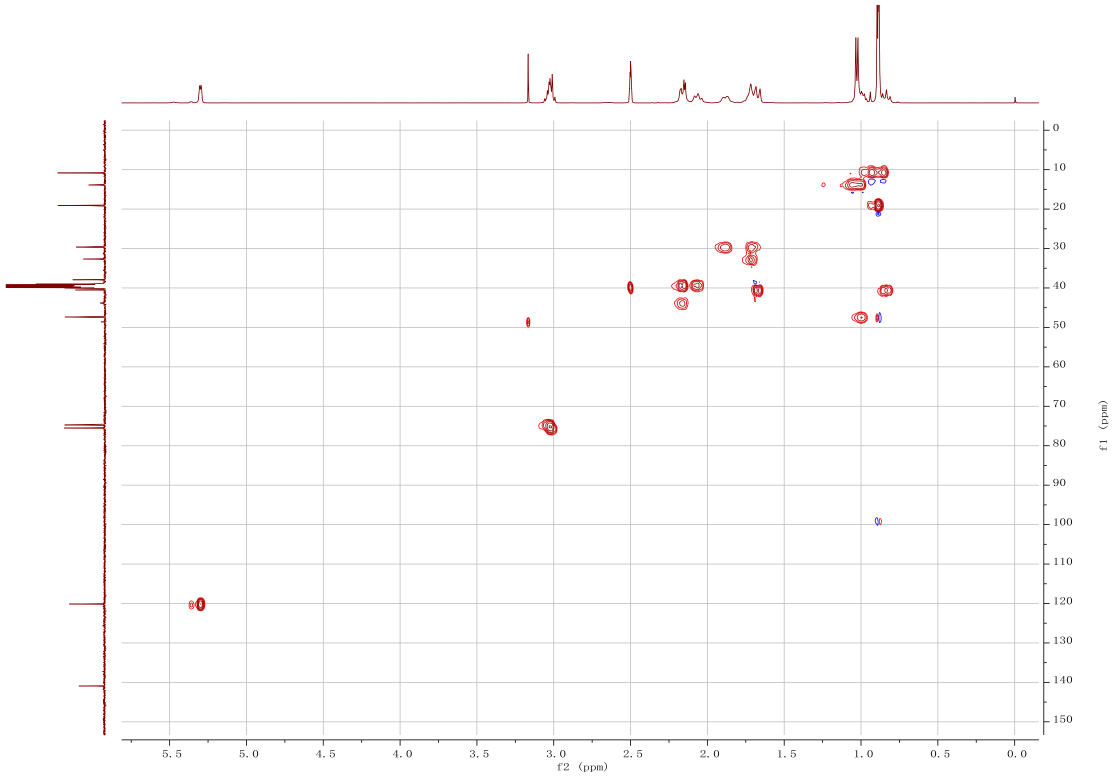

Figure S13. HSQC spectrum of **2** in DMSO-d_6.

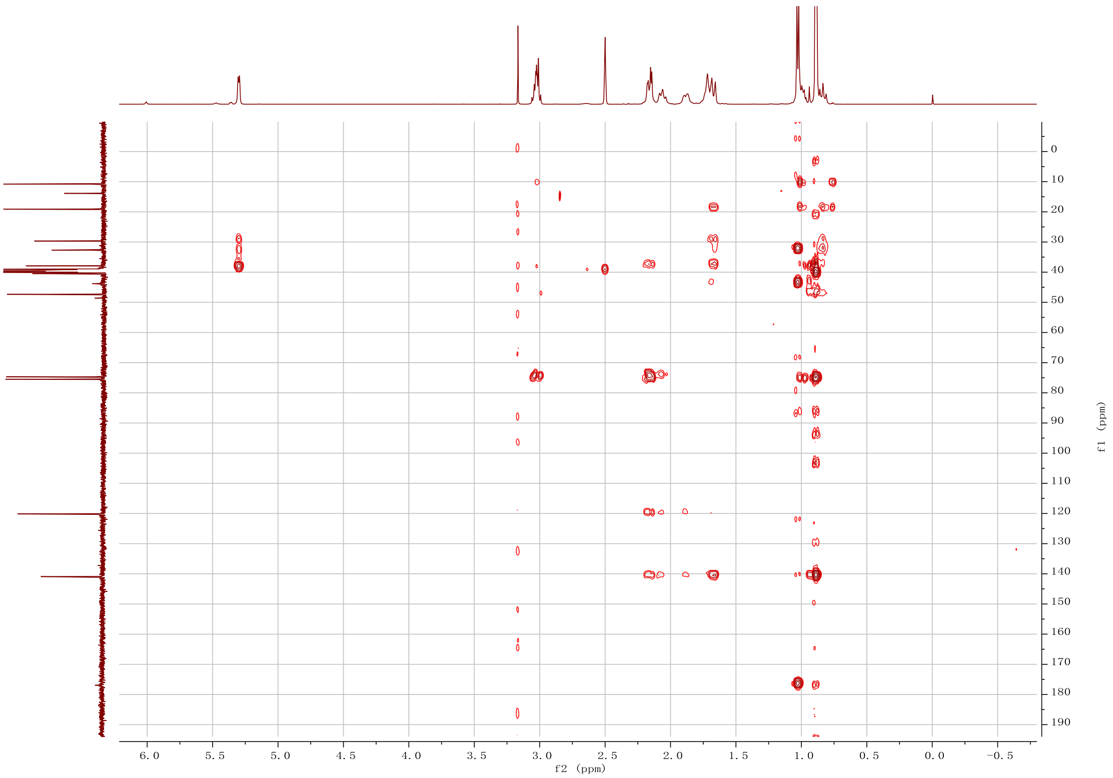

Figure S14. HMBC spectrum of **2** in DMSO-d_6.

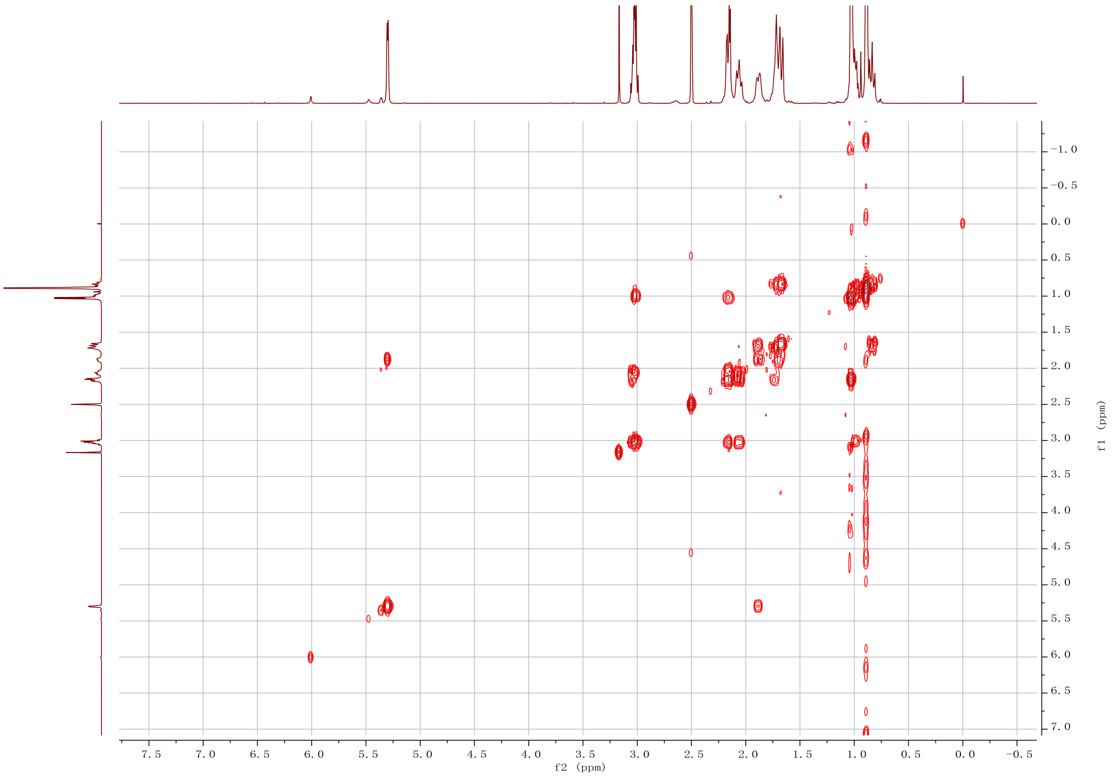

Figure S15. ^1H–^1H COSY spectrum of **2** in DMSO-d_6.

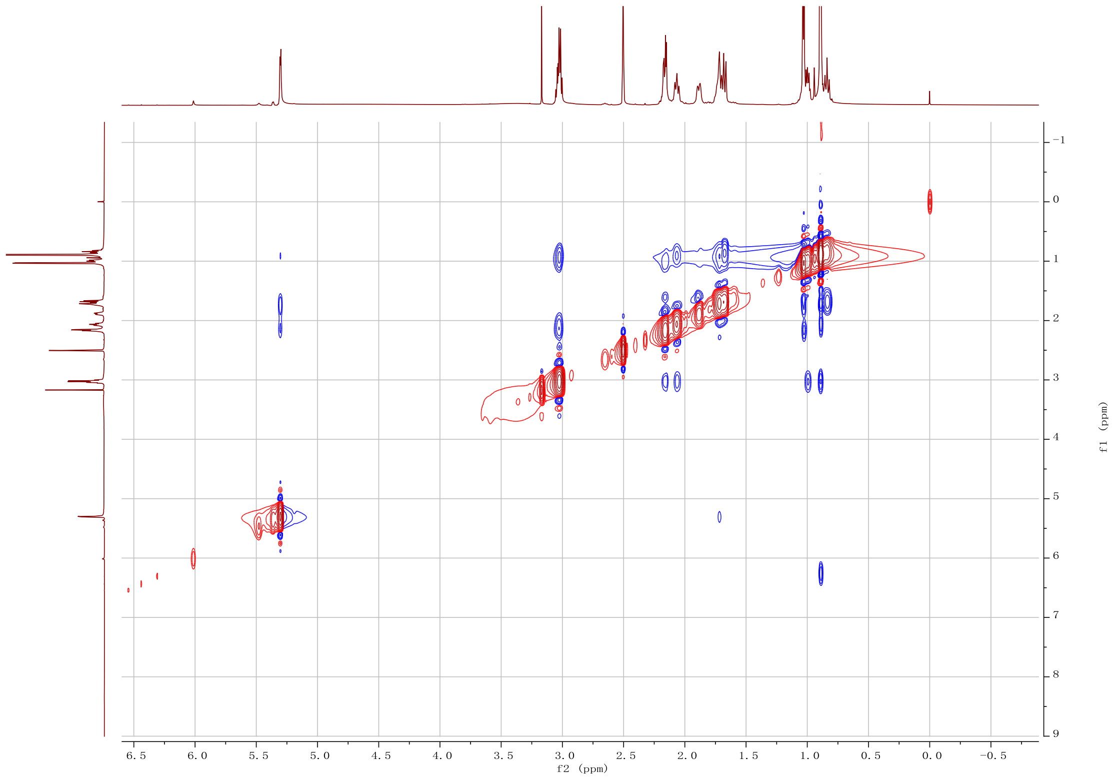

Figure S16. NOESY spectrum of **2** in DMSO-d_6.

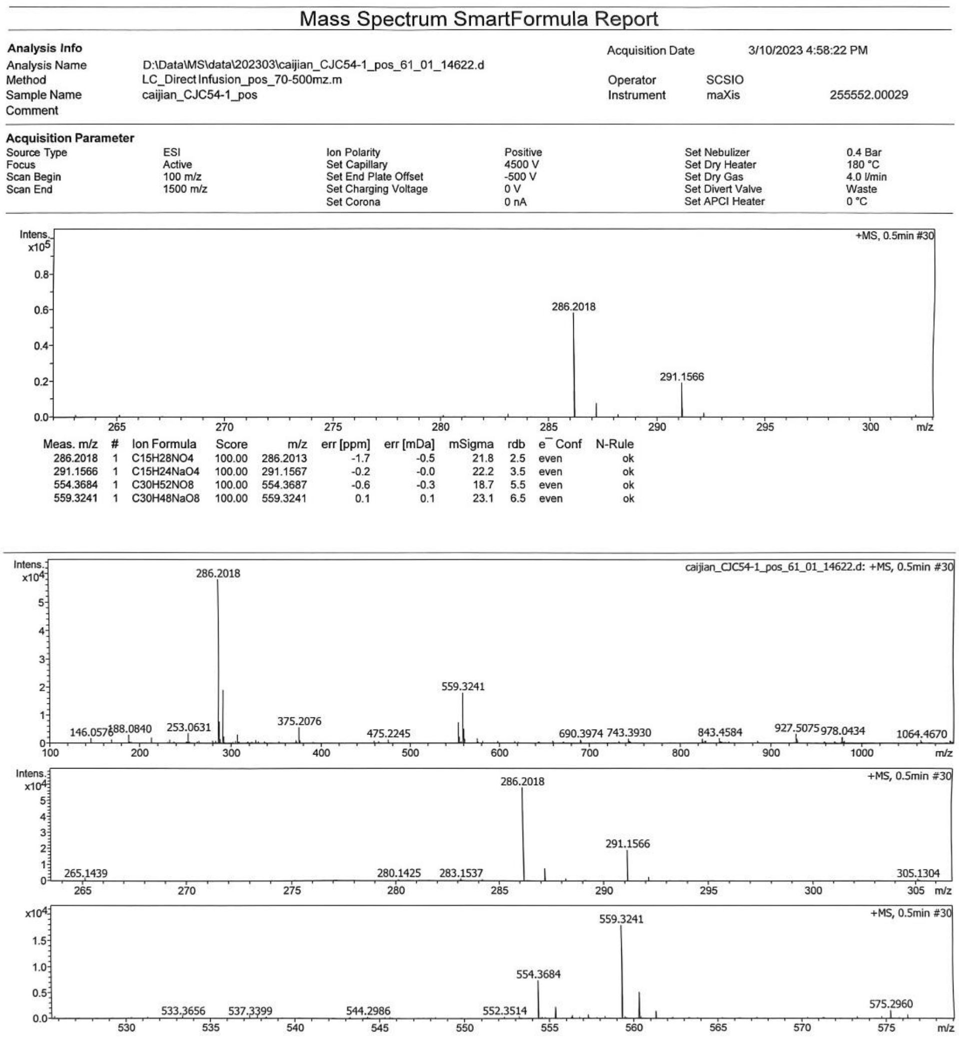

Meas. m/z	#	Ion Formula	Score	m/z	err [ppm]	err [mDa]	mSigma	rdb	e⁻ Conf	N-Rule
286.2018	1	C15H28NO4	100.00	286.2013	-1.7	-0.5	21.8	2.5	even	ok
291.1566	1	C15H24NaO4	100.00	291.1567	-0.2	-0.0	22.2	3.5	even	ok
554.3684	1	C30H52NO8	100.00	554.3687	-0.6	-0.3	18.7	5.5	even	ok
559.3241	1	C30H48NaO8	100.00	559.3241	0.1	0.1	23.1	6.5	even	ok

Figure S17. HRESIMS spectrum of **2**.

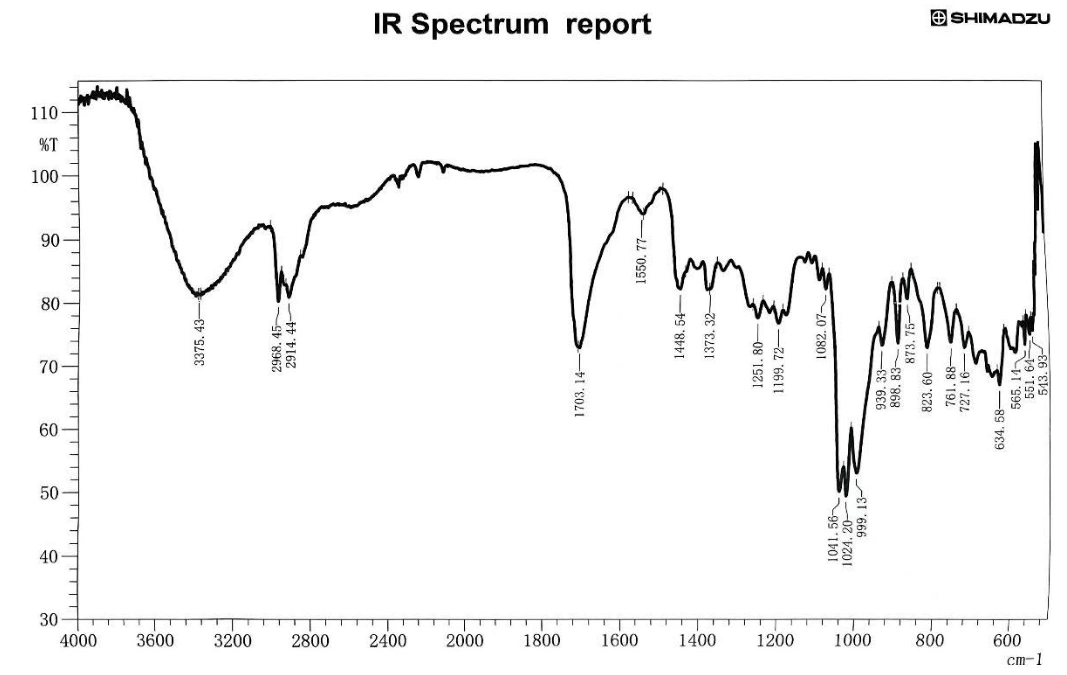

Figure S18. IR spectrum of **2**.

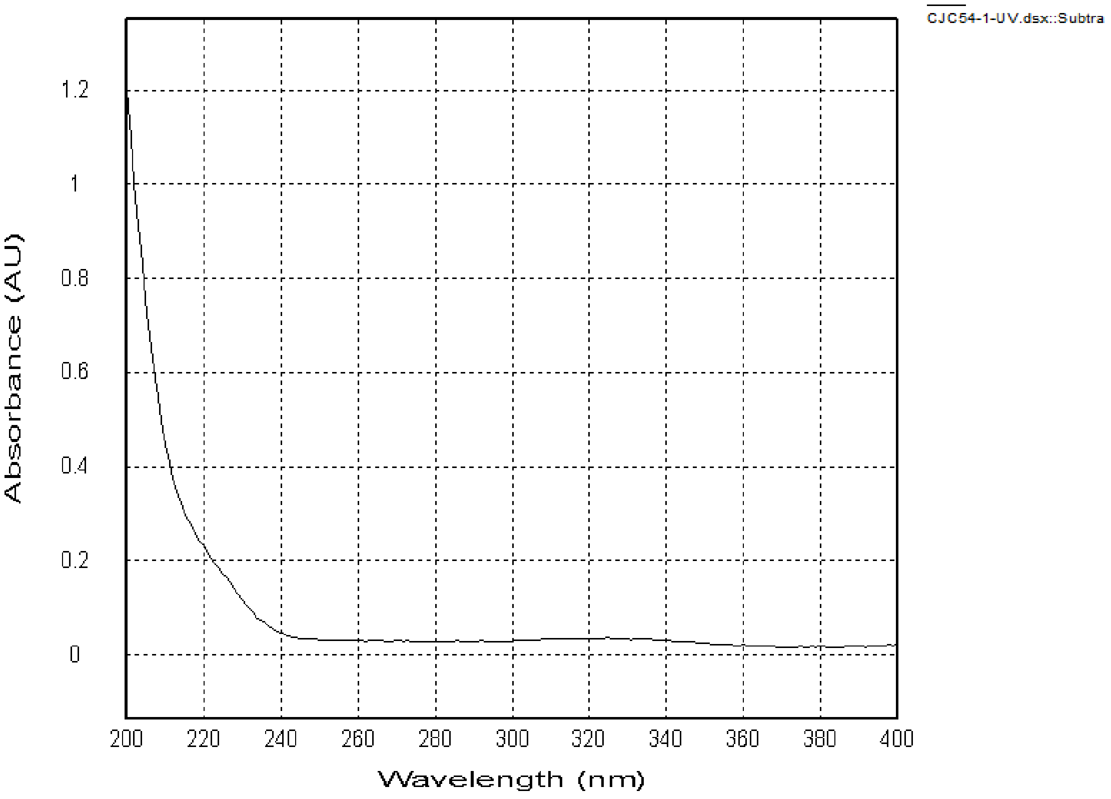

Figure S19. UV spectrum of **2** in MeOH.

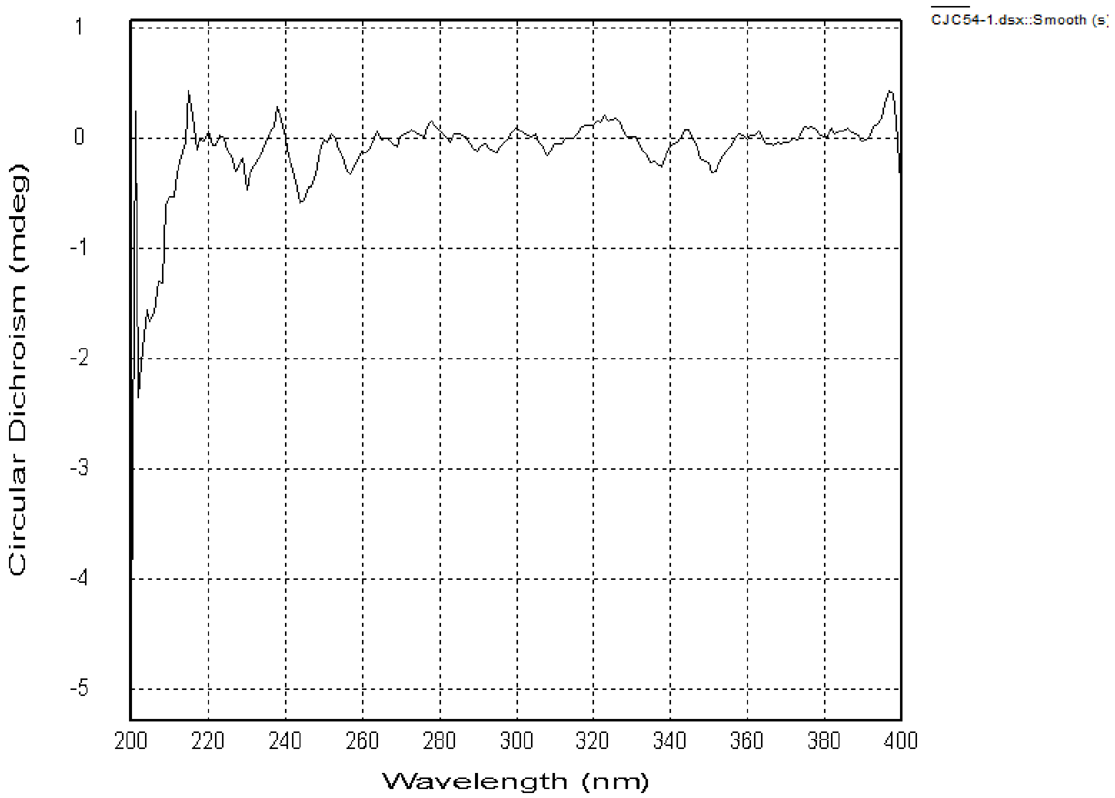

Figure S20. ECD spectrum of **2** in MeOH.

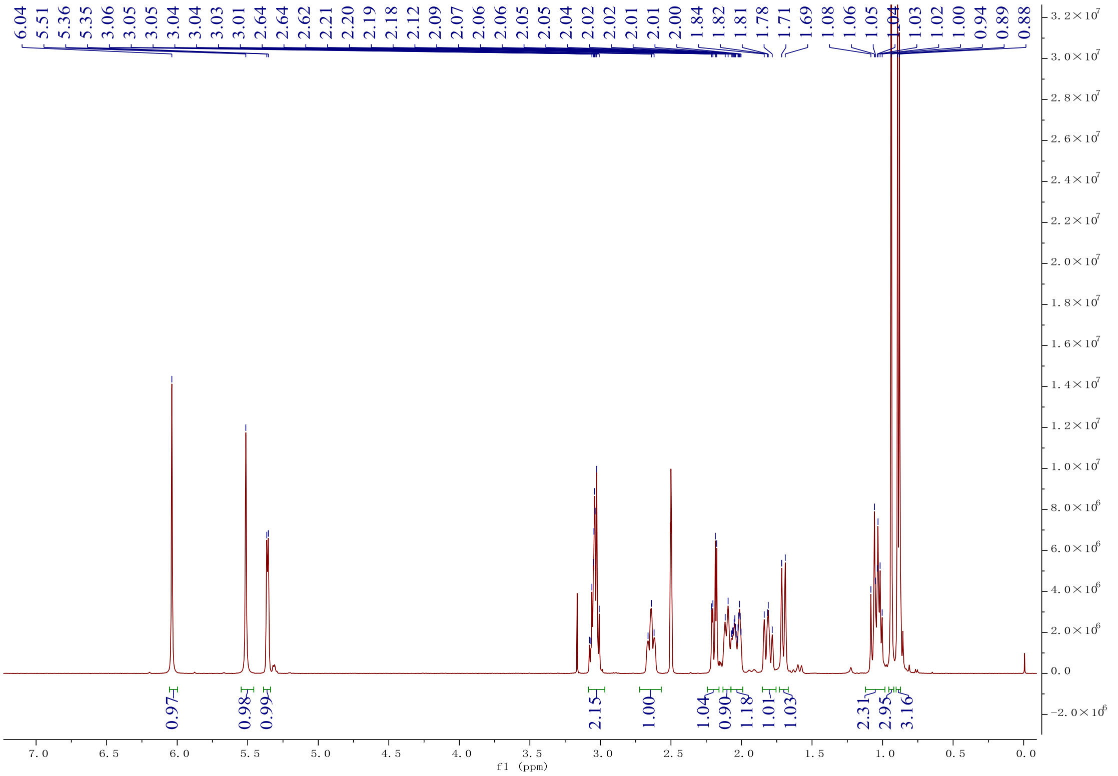

Figure S21. ^{1}H NMR (500 MHz) spectrum of **3** in DMSO-d_6.

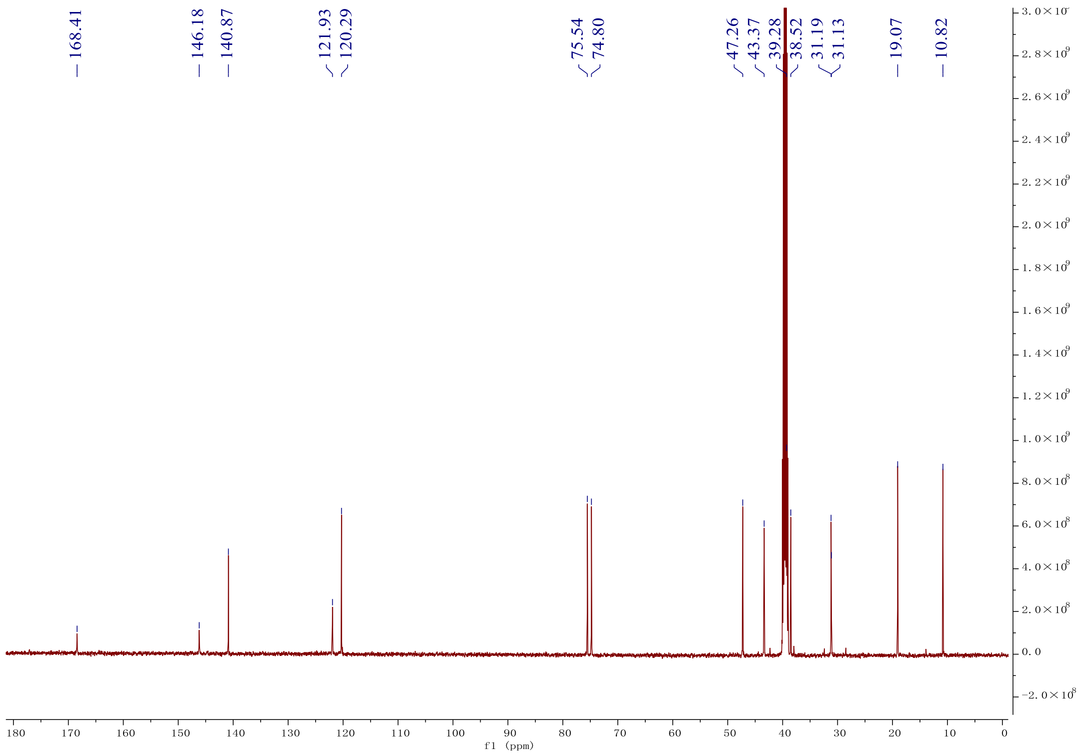

Figure S22. ^{13}C NMR (125 MHz) spectrum of **3** in DMSO-d_6.

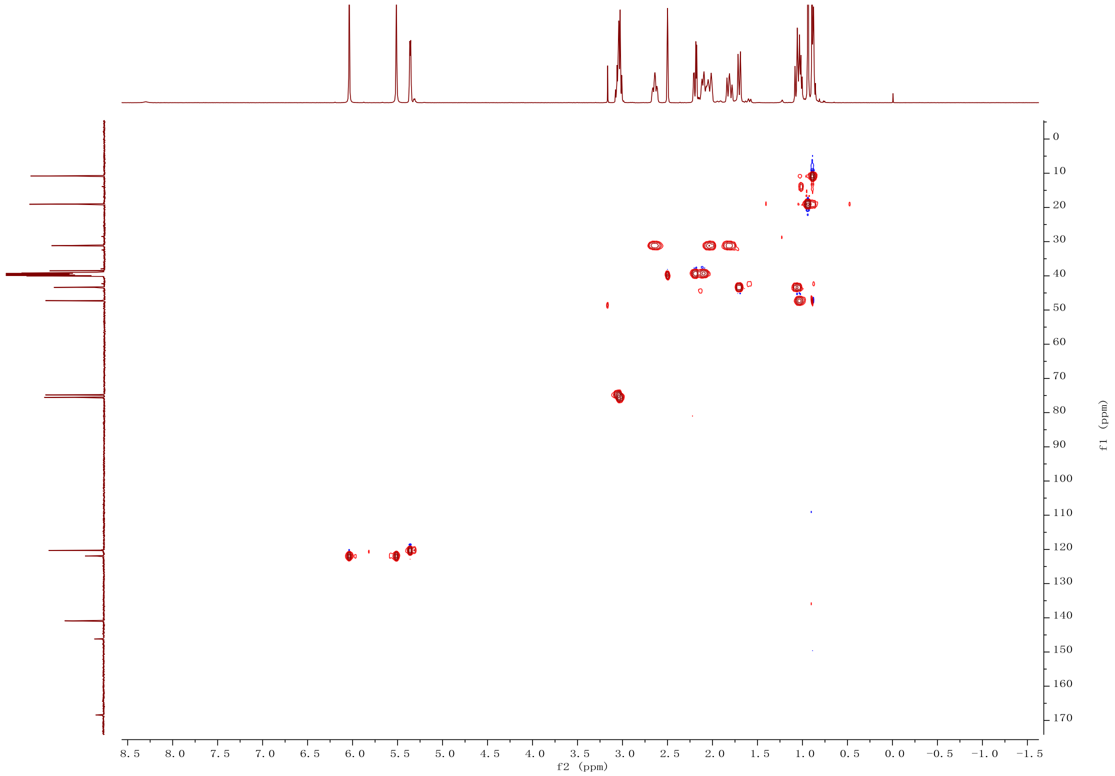

Figure S23. HSQC spectrum of **3** in DMSO-d_6.

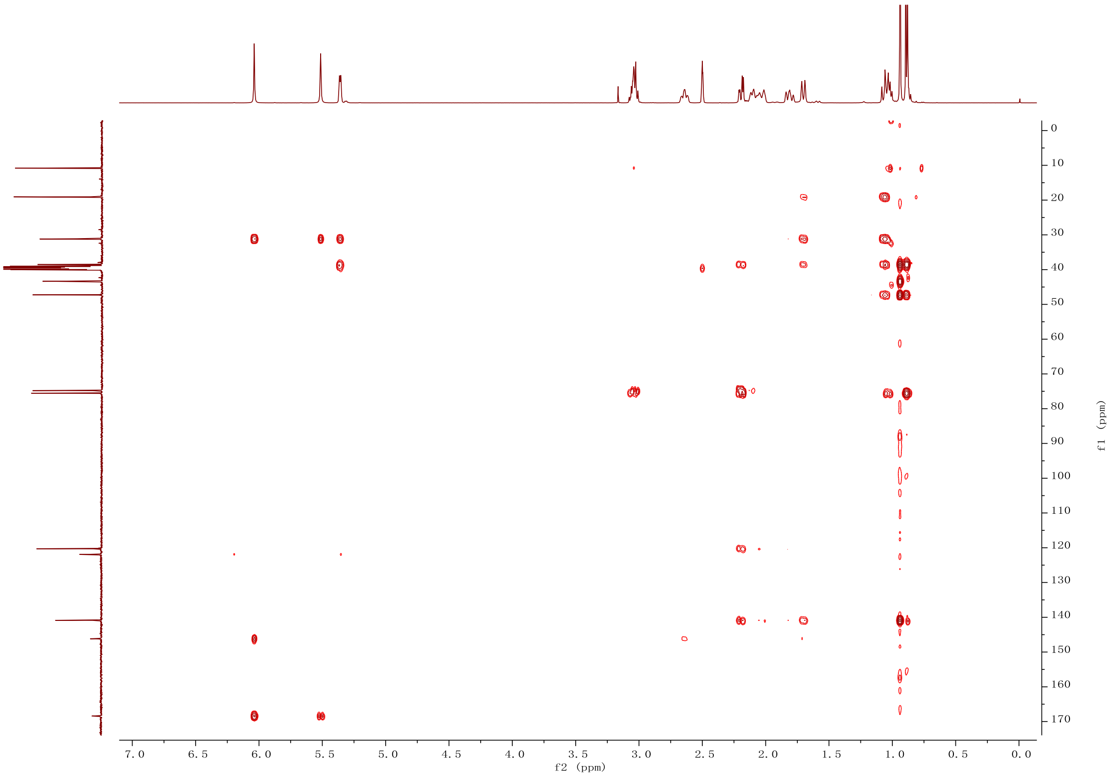

Figure S24. HMBC spectrum of **3** in DMSO-d_6.

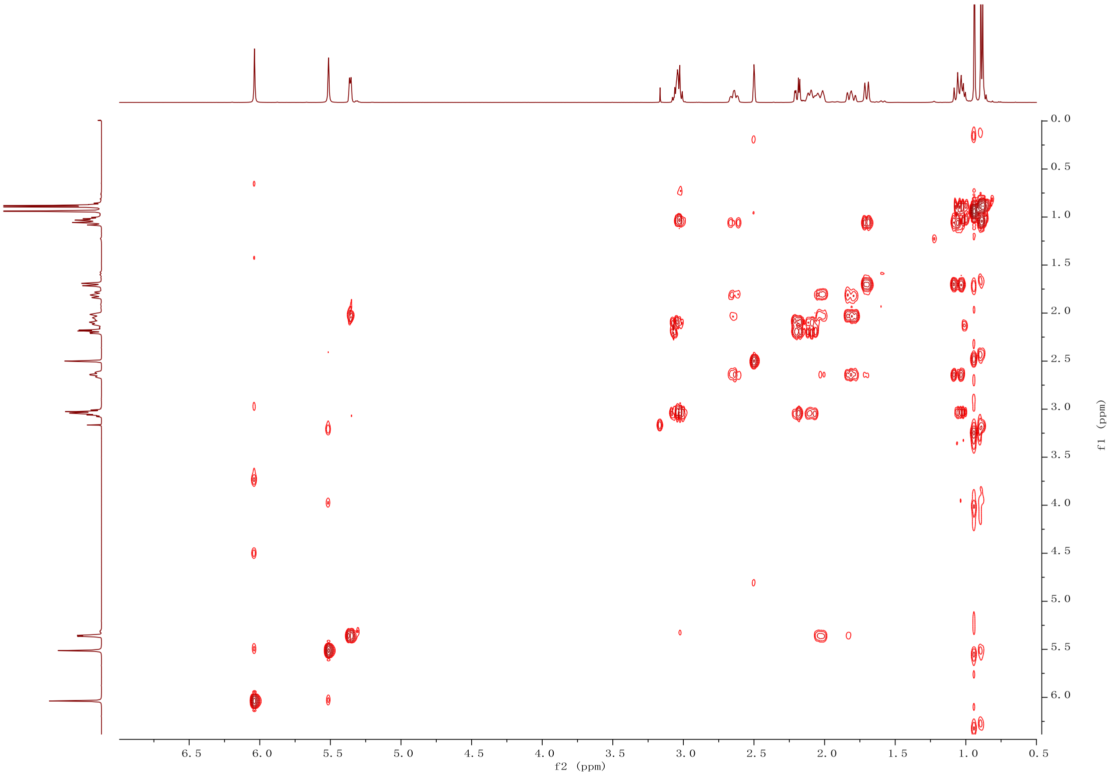

Figure S25. ^{1}H–^{1}H COSY spectrum of **3** in DMSO-d_6.

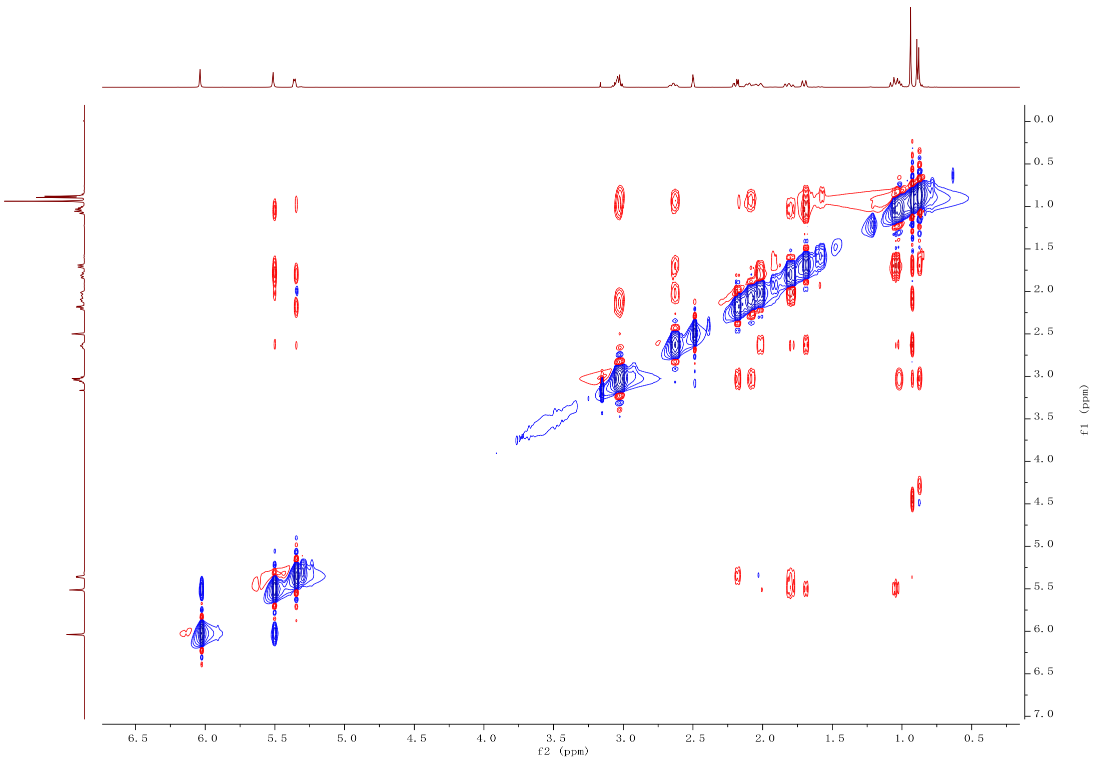

Figure S26. NOESY spectrum of **3** in DMSO-d_6.

Mass Spectrum SmartFormula Report

Analysis Info

Analysis Name	D:\Data\MS\data\202302\caijian_CJC54-2_neg_1_01_14382.d	Acquisition Date	2/9/2023 4:02:16 PM
Method	LC_Direct Infusion_neg_70-500mz.m	Operator	SCSIO
Sample Name	caijian_CJC 54-2_neg	Instrument	maXis 255552.00029
Comment			

Acquisition Parameter

Source Type	ESI	Ion Polarity	Negative	Set Nebulizer	0.4 Bar
Focus	Active	Set Capillary	4200 V	Set Dry Heater	180 °C
Scan Begin	100 m/z	Set End Plate Offset	-500 V	Set Dry Gas	4.0 l/min
Scan End	1500 m/z	Set Charging Voltage	0 V	Set Divert Valve	Waste
		Set Corona	0 nA	Set APCI Heater	0 °C

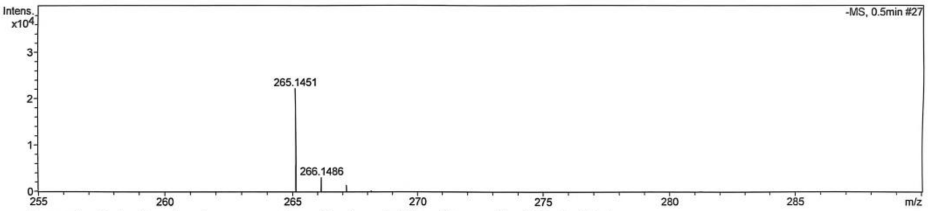

Meas. m/z	#	Ion Formula	Score	m/z	err [ppm]	err [mDa]	mSigma	rdb	e⁻ Conf	N-Rule
265.1451	1	C15H21O4	100.00	265.1445	2.2	0.6	19.0	5.5	even	ok
531.2972	1	C30H43O8	100.00	531.2963	1.6	0.8	109.5	9.5	even	ok

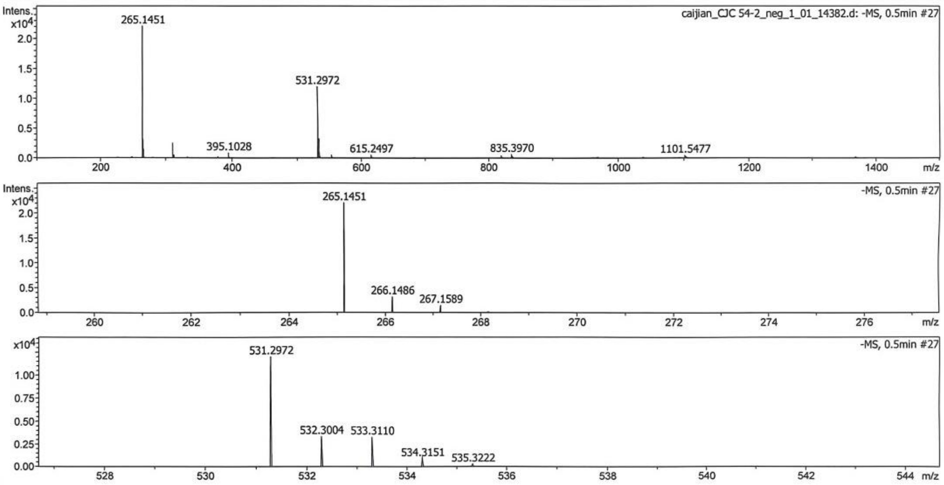

Figure S27. HRESIMS spectrum of **3**.

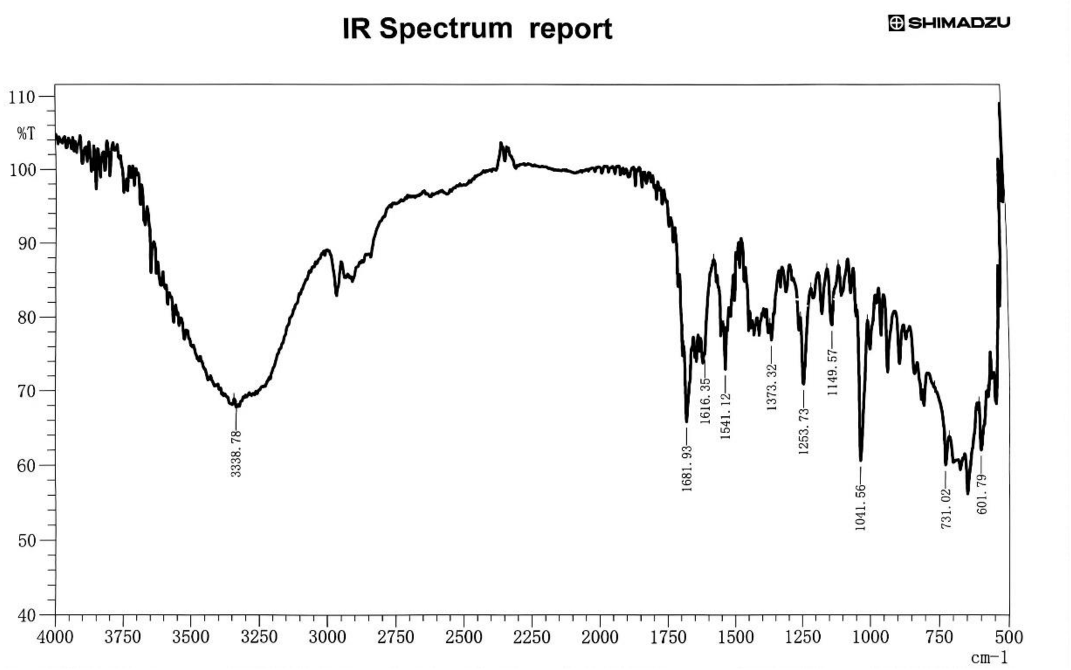

Figure S28. IR spectrum of **3**.

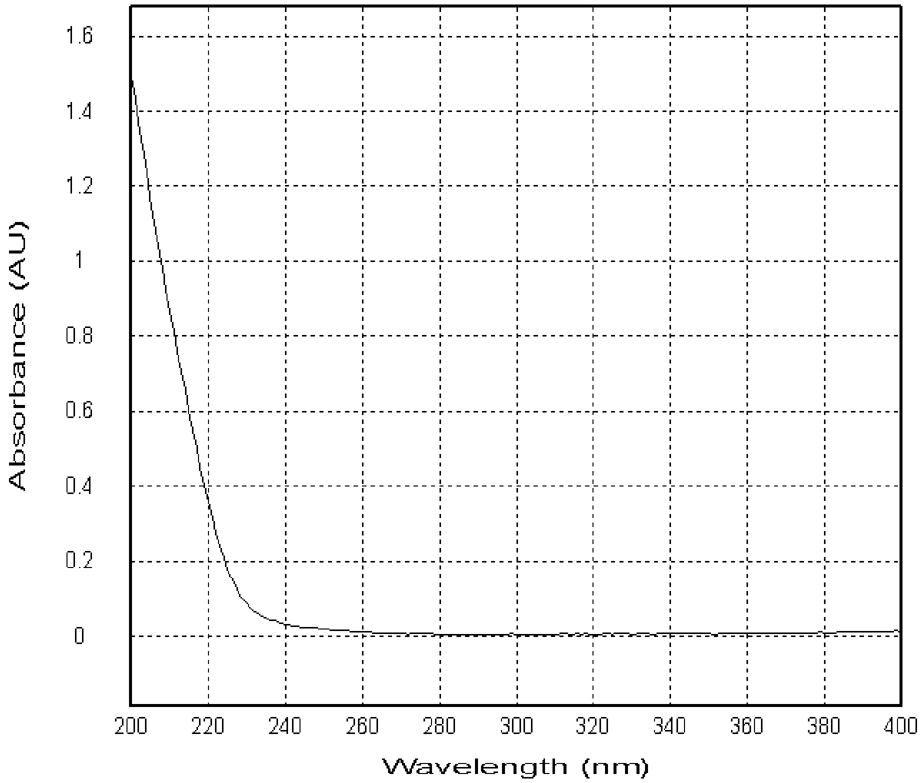

Figure S29. UV spectrum of **3** in MeOH.

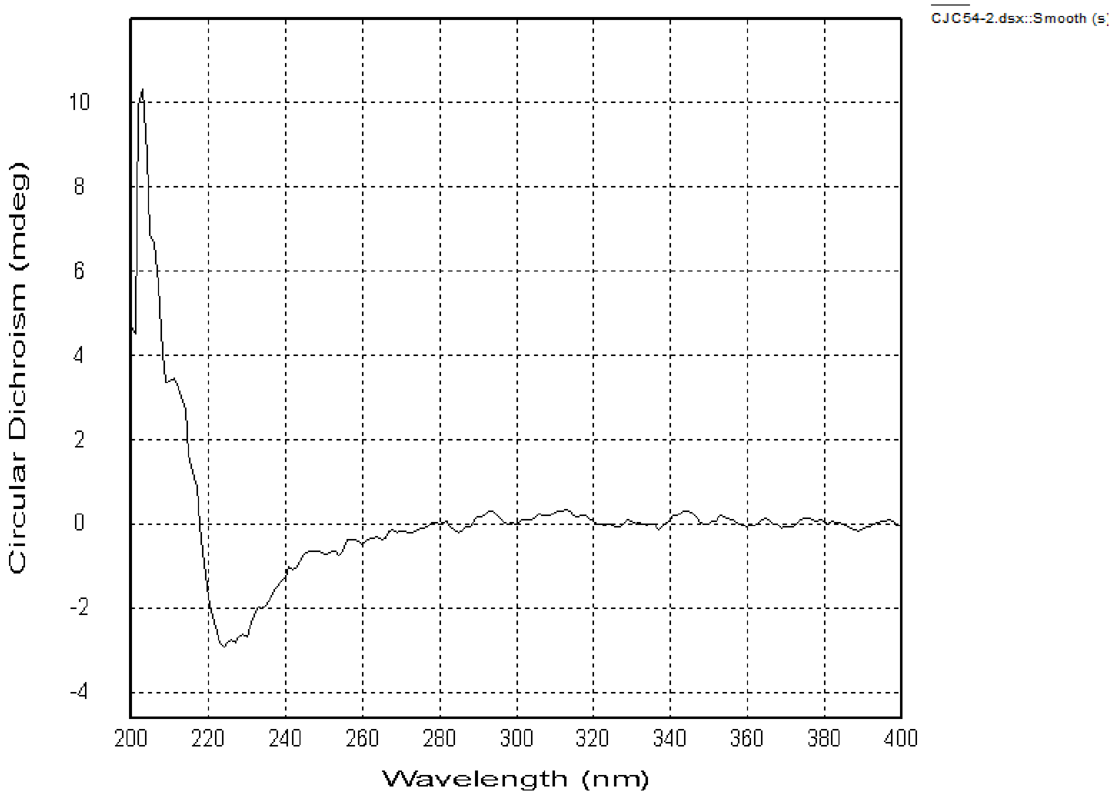

Figure S30. ECD spectrum of **3** in MeOH.

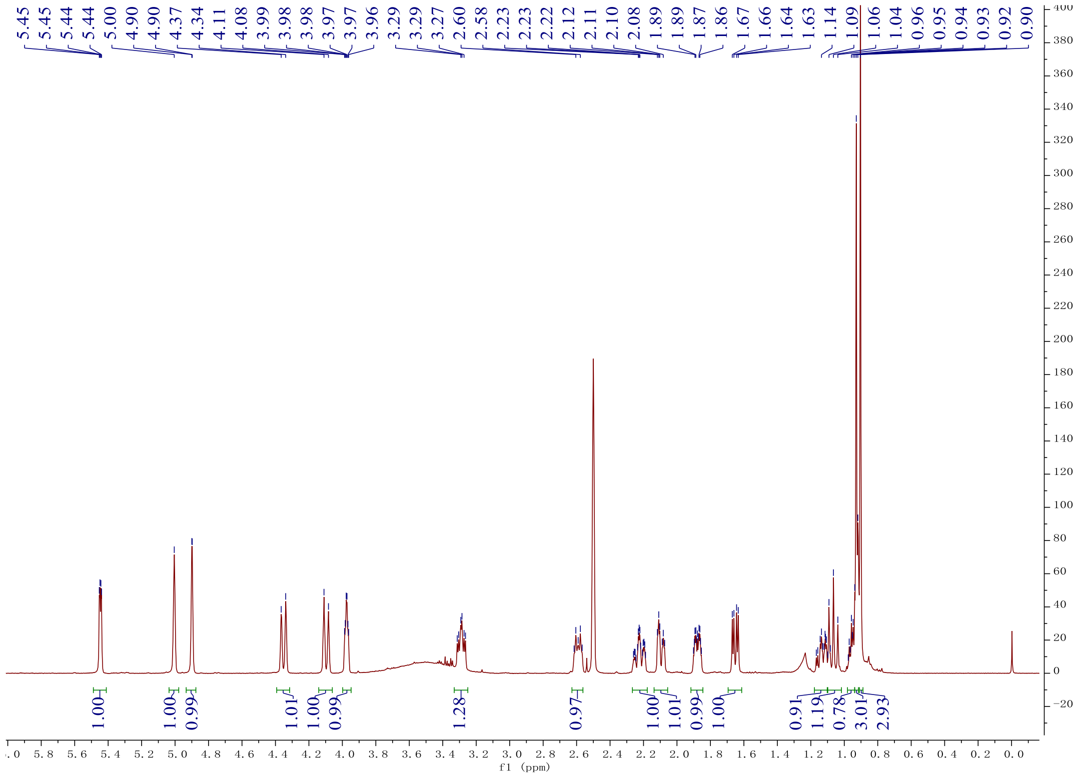

Figure S31. ^{1}H NMR (500 MHz) spectrum of **4** in DMSO-d_6.

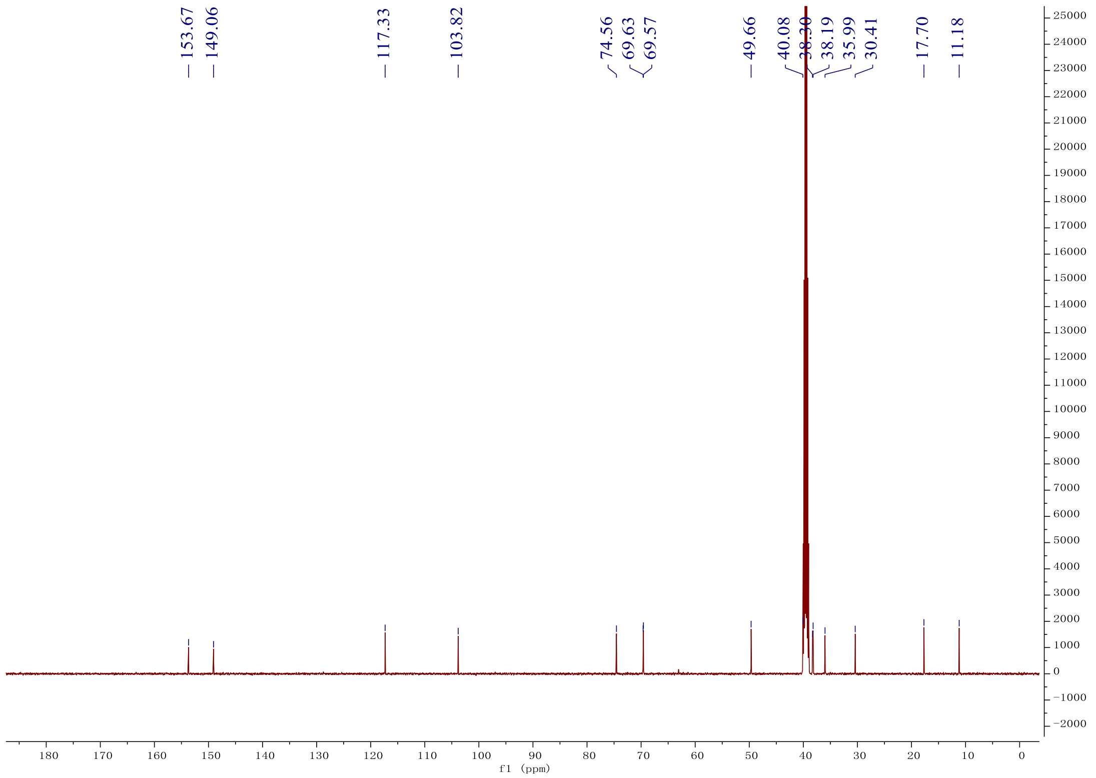

Figure S32. ^{13}C NMR (125 MHz) spectrum of **4** in DMSO-d_6.

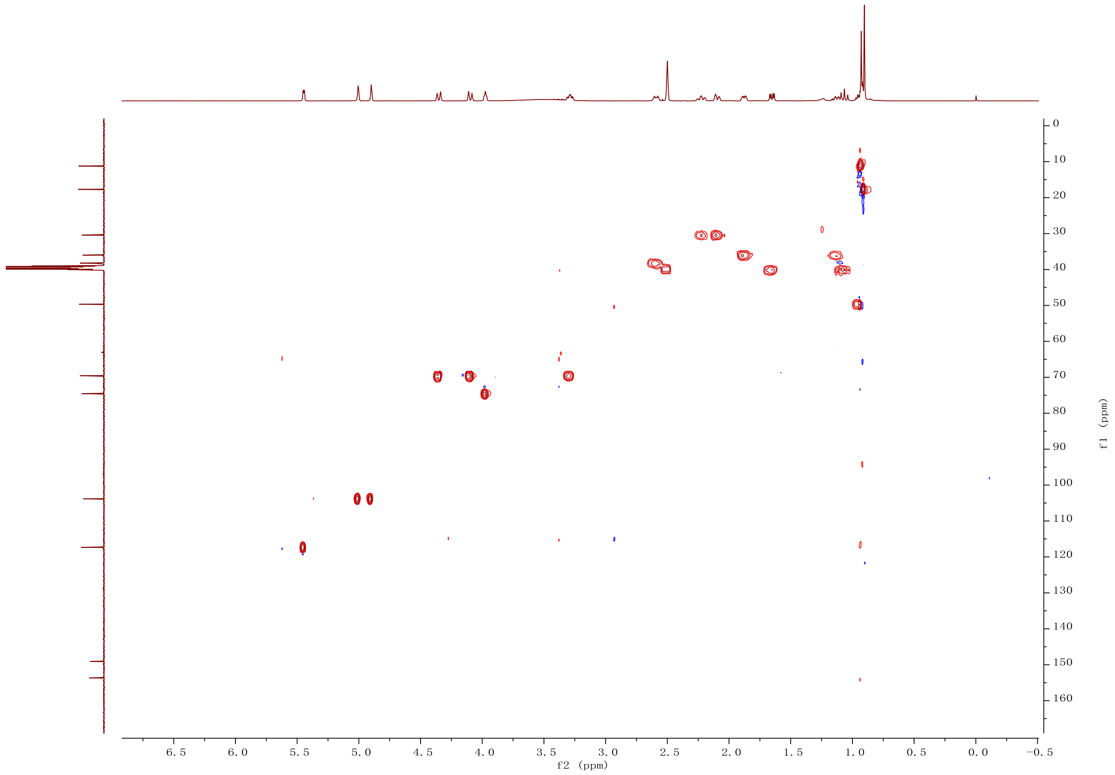

Figure S33. HSQC spectrum of **4** in DMSO-d_6.

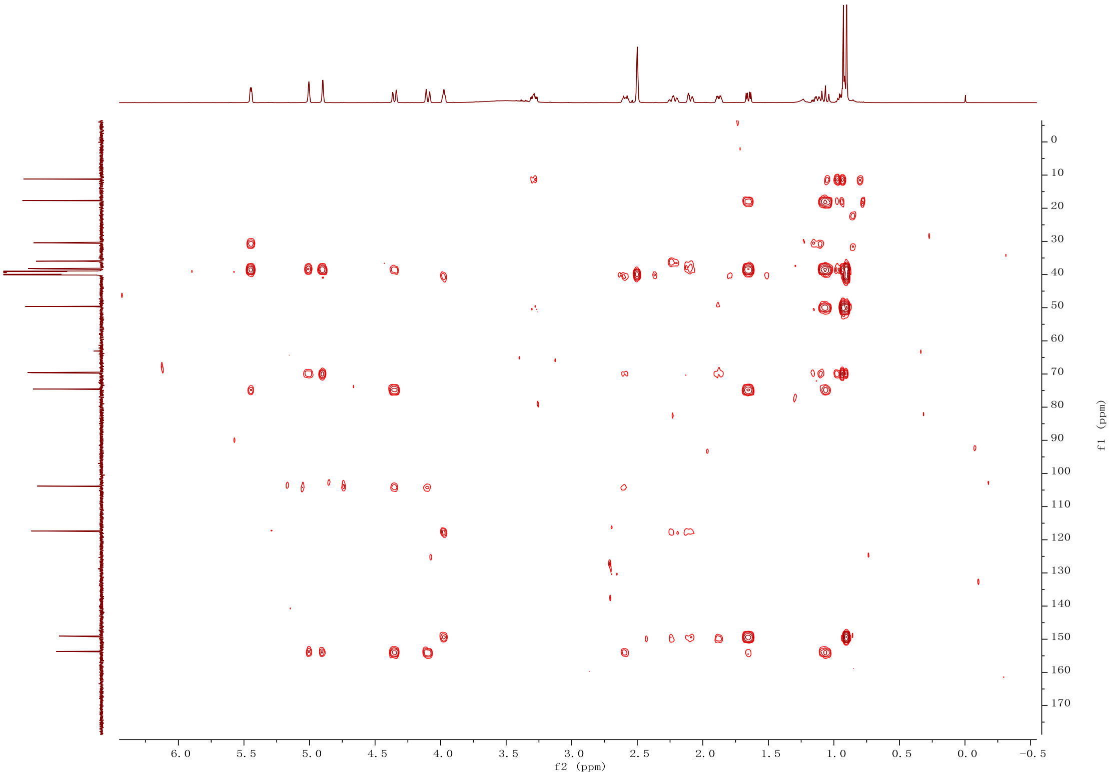

Figure S34. HMBC spectrum of **4** in DMSO-d_6.

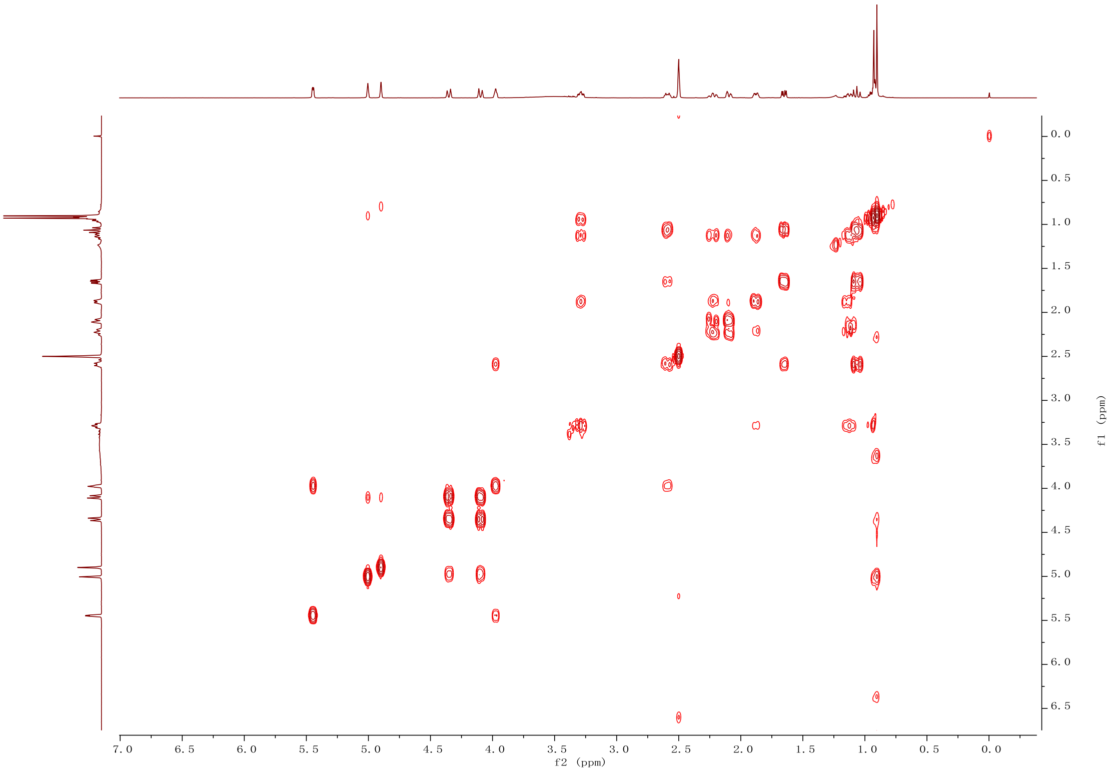

Figure S35. ^{1}H-^{1}H COSY spectrum of **4** in DMSO-d_6.

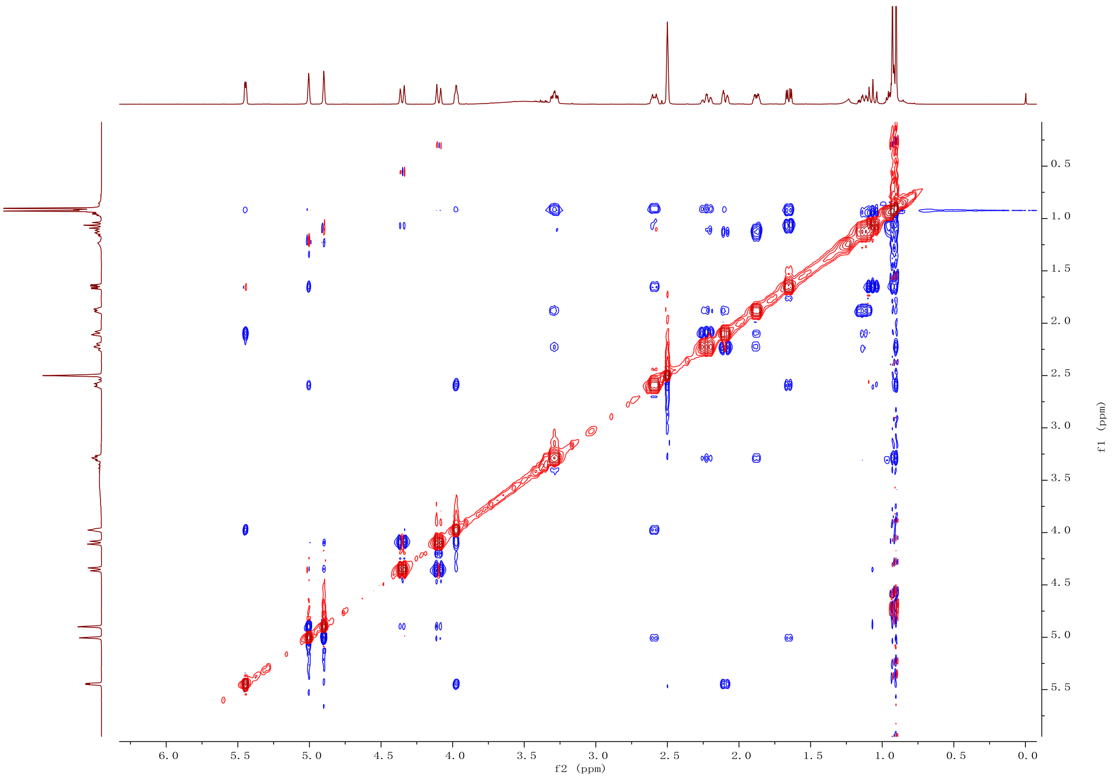

Figure S36. NOESY spectrum of **4** in DMSO-d_6.

•

Mass Spectrum SmartFormula Report

Analysis Info			
Analysis Name	D:\Data\MS\data\202302\caijian_CJC62_pos_93_01_14364.d	Acquisition Date	2/7/2023 3:42:44 PM
Method	LC_Direct Infusion_pos_70-500mz.m	Operator	SCSIO
Sample Name	caijian_CJC 62_pos	Instrument	maXis 255552.00029
Comment			

Acquisition Parameter					
Source Type	ESI	Ion Polarity	Positive	Set Nebulizer	0.4 Bar
Focus	Active	Set Capillary	4500 V	Set Dry Heater	180 °C
Scan Begin	100 m/z	Set End Plate Offset	-500 V	Set Dry Gas	4.0 l/min
Scan End	1500 m/z	Set Charging Voltage	0 V	Set Divert Valve	Waste
		Set Corona	0 nA	Set APCI Heater	0 °C

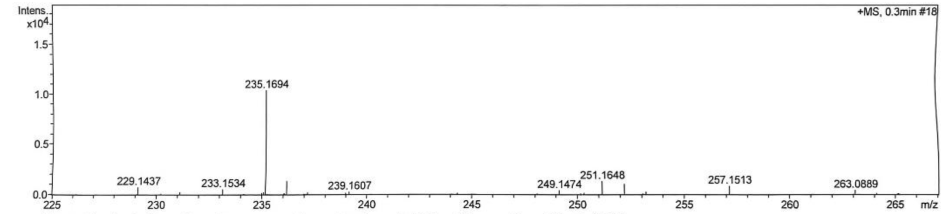

Meas. m/z	#	Ion Formula	Score	m/z	err [ppm]	err [mDa]	mSigma	rdb	e⁻ Conf	N-Rule
235.1694	1	C15H23O2	100.00	235.1693	0.4	0.1	21.0	4.5	even	ok
252.1958	1	C15H26NO2	100.00	252.1958	-0.1	-0.0	52.7	3.5	even	ok
257.1513	1	C15H22NaO2	100.00	257.1512	0.4	0.1	36.8	4.5	even	ok

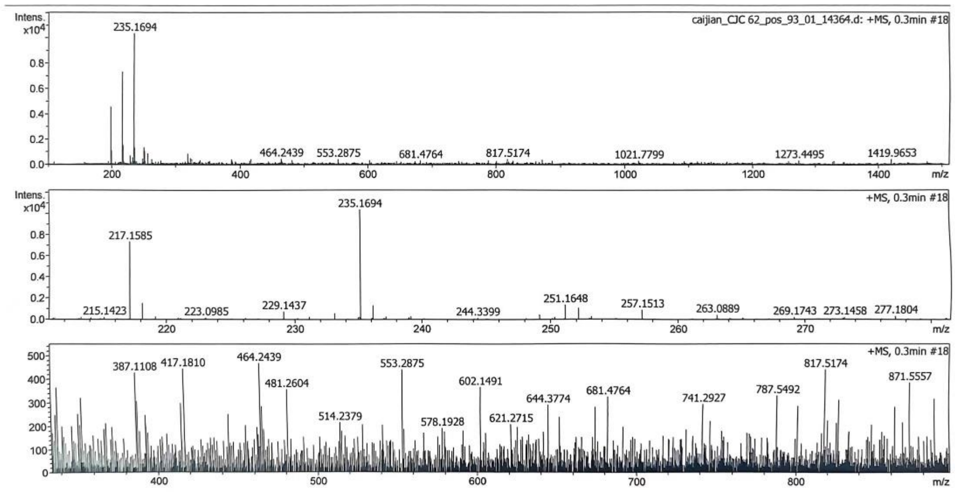

Figure S37. HRESIMS spectrum of **4**.

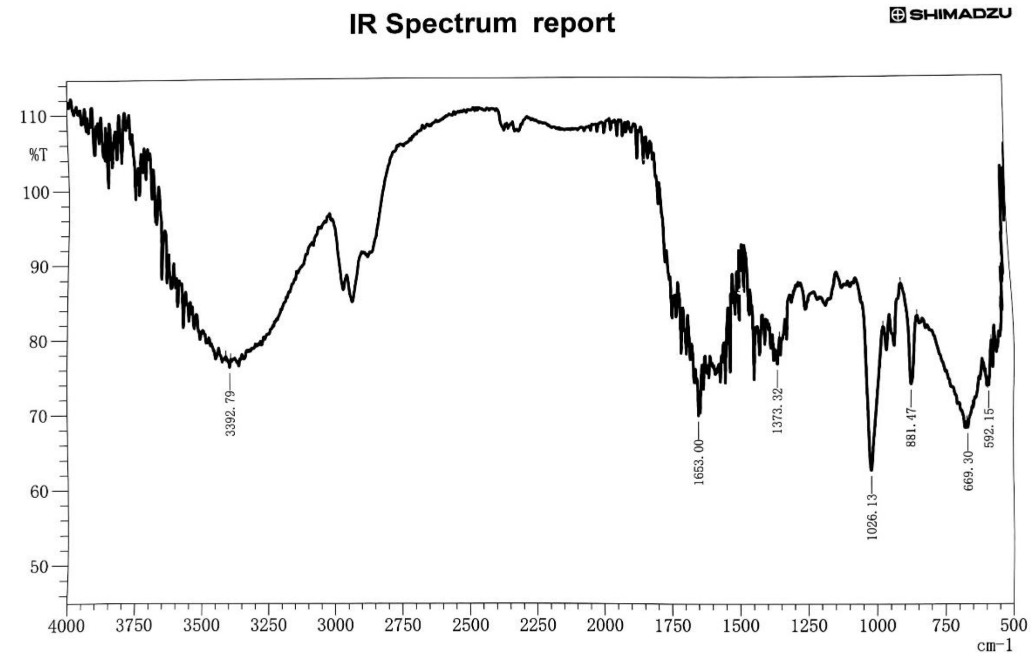

Figure S38. IR spectrum of **4**.

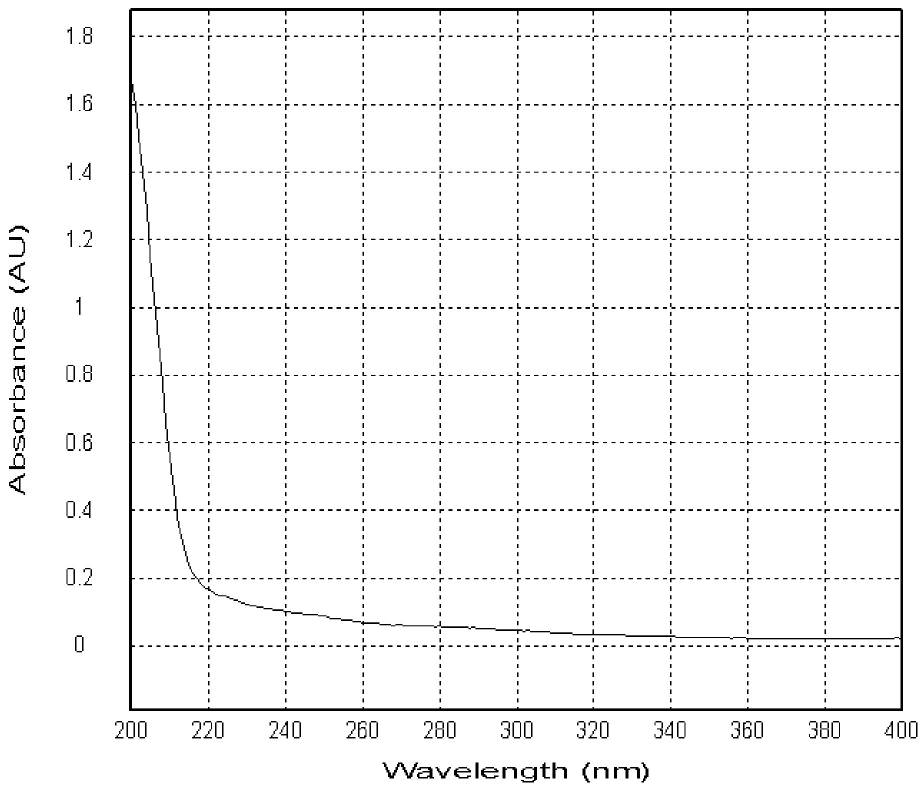

Figure S39. UV spectrum of **4** in MeOH.

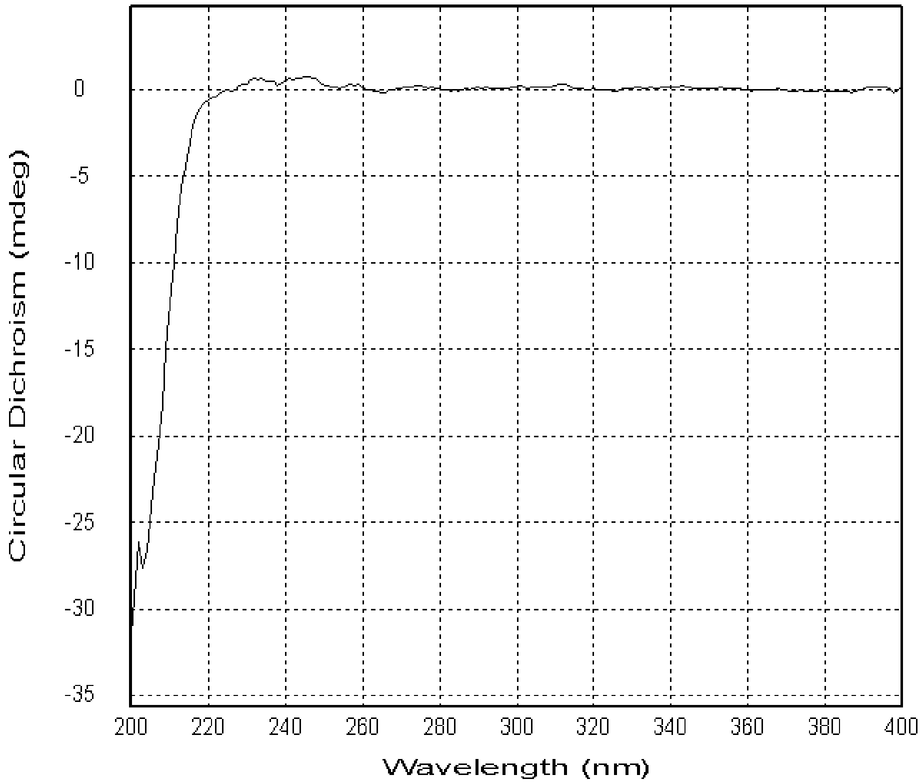

Figure S40. ECD spectrum of **4** in MeOH.

Table S1. Primers of RT-qPCR

Primer	Sequence (5'→3')
H-*RORα*-F	CACGACGACCTCAGTAACTACA
H-*RORα*-R	TGGTGAACGAACAGTAGGGAA
H-*BMAL1*-F	AAATCGCTTTGAGGTGAC
H-*BMAL1*-R	CTTTCCGTTTGCGGTTGC
H-*CLOCK*-F	TGCGAGGAACAATAGACCCAA
H-*CLOCK*-R	ATGGCCTATGTGTGCGTTGTA
H-*LXRα*-F	GCTGCAAGTGGAATTCATCAACC
H-*LXRα*-R	ATATGTGTGCTGCAGCCTCTCCA
H-*ABCA1*-F	ACCCACCCTATGAACAACATGA
H-*ABCA1*-R	GAGTCGGGTAACGGAAACAGG
H-*PPARα*-F	TTCGCAATCCATCGGCGAG
H-*PPARα*-R	CCACAGGATAAGTCACCGAGG
H-*ACOX1*-F	ACTCGCAGCCAGCGTTATG
H-*ACOX1*-R	AGGGTCAGCGATGCCAAAC
H-*CPT1α*-F	ATCAATCGGACTCTGGAAACGG
H-*CPT1α*-R	TCAGGGAGTAGCGCATGGT
H-*GAPDH*-F	GCTCTCTGCTCCTCCTGTTC
H-*GAPDH*-R	ACGACCAAATCCGTTGACTC
H-*FXR*-F	GGCTCGGGGATACTGGATACA
H-*FXR*-R	CTGGCATGAAGCGTTGTCC
H-*CYP7A1*-F	CAACGTATCATGAGACCTCCAGTC
H-*CYP7A1*-R	CAGCTTCAAACATCACTCGGTAG

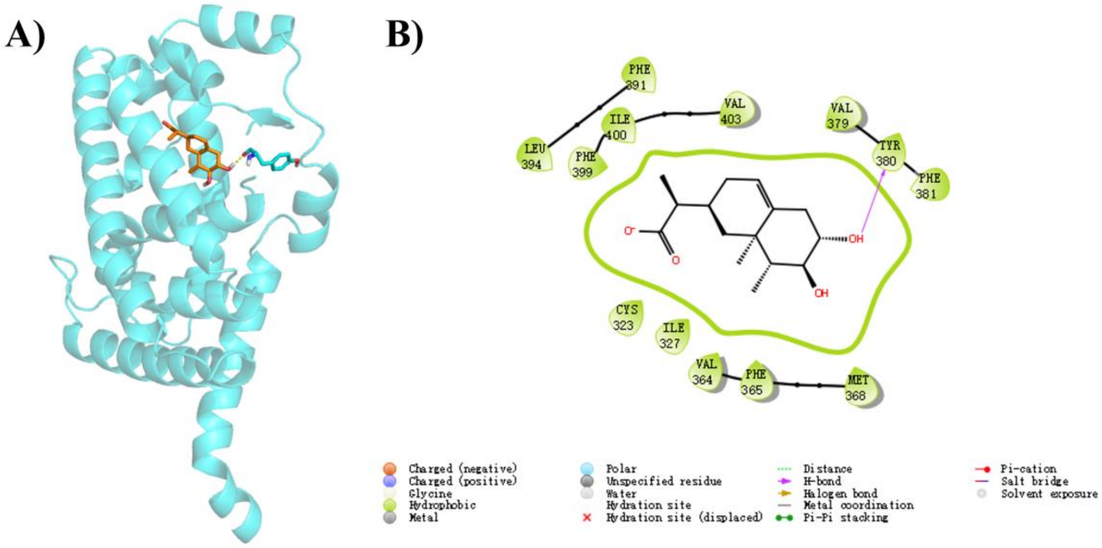

Figure S41. The docking result of **1** with RORα.A) 3D and B) 2D binding mode of **1** with RORα (PDB code:1N83) predicted by *in silico* molecular docking.

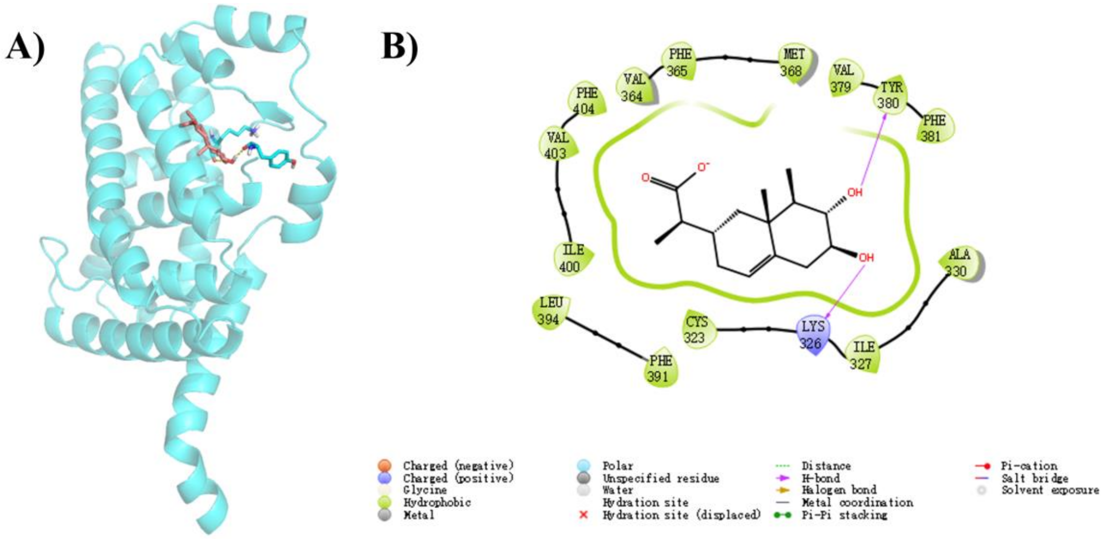

Figure S42. The docking result of **2** with RORα. A) 3D and B) 2D binding mode of **2** with RORα (PDB code:1N83) predicted by *in silico* molecular docking.

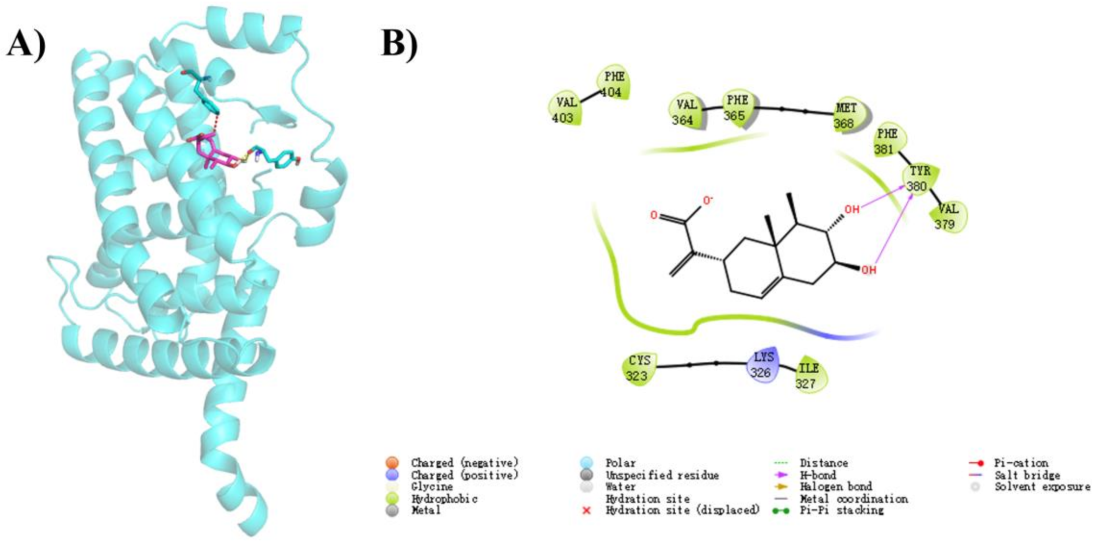

Figure S43. The docking result of **3** with RORα. A) 3D (Yellow bond:H-bond; Red bond:bad contact) and B) 2D binding mode of **3** with RORα (PDB code:1N83) predicted by in silico molecular docking.

Table S2. Energies of **4** at B3LYP/6–311g (d, p) level.

Configuration	Conformer	E (Hartree)	E (kcal/mol)	Population
3*R*, 4*R*, 5*R*, 7*S*, 8*S*	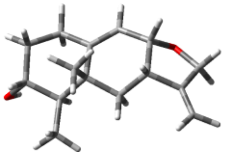 1	-735.220313554	-461358.098958271	28.15%
3*R*, 4*R*, 5*R*, 7*S*, 8*S*	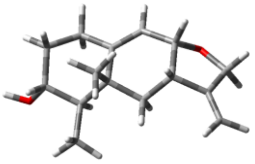 2	-735.220802572	-461358.405821956	47.26%
3*R*, 4*R*, 5*R*, 7*S*, 8*S*	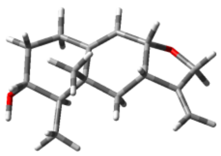 3	-735.220185619	-461358.018677779	24.59%
3*S*, 4*S*, 5*S*, 7*R*, 8*R*	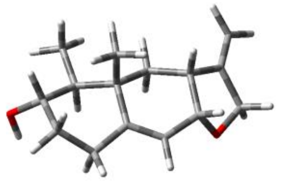 1	-735.220313555	-461358.098958898	28.16%
3*S*, 4*S*, 5*S*, 7*R*, 8*R*	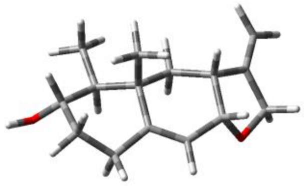 2	-735.220802527	-461358.405793718	47.26%
3*S*, 4*S*, 5*S*, 7*R*, 8*R*	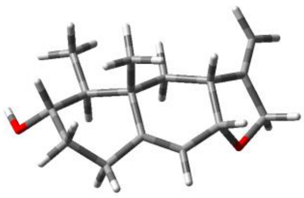 3	-735.220185592	-461358.018660836	24.59%
